# Machine Learning-Driven Prediction and Design Guidance for Asphalt Concrete Using Marshall Stability and Indirect Tensile Strength

**DOI:** 10.3390/ma19163408

**Published:** 2026-08-11

**Authors:** Jianglei Xing, Xiao Tan, Mu Guo, Pengwei Guo, Yuhuan Wang, Dongzhao Jin

**Affiliations:** 1 College of Water Conservancy and Hydropower Engineering, Hohai University, Nanjing 210024, China; 241602010139@hhu.edu.cn; 2 State Key Laboratory of Water Disaster Prevention, Nanjing 210024, China; 3 Shanghai Research Institute of Building Science Co., Ltd., Shanghai 201108, China; guomu@sribs.com.cn; 4 Bartlett School of Sustainable Construction, University College London, London WC1E 6BT, UK; pengwei.guo@ucl.ac.uk; 5 Department of Civil, Environmental, and Architectural Engineering, University of Colorado Boulder, Boulder, CO 80309, USA; 6 Department of Civil, Environmental, and Geospatial Engineering, Michigan Technological University, Houghton, MI 49931, USA

**Keywords:** asphalt concrete, Marshall stability, indirect tensile strength, machine learning, Pareto filtering, TOPSIS ranking

## Abstract

This work addresses the simultaneous prediction of Marshall Stability (MS) and Indirect Tensile Strength (ITS) by integrating machine learning models with multi-objective optimization for the preliminary design of asphalt concrete. Based on 389 experimental samples, 15 variables were selected to describe asphalt properties, aggregate gradation, volumetric parameters and fiber characteristics, and four dual-output prediction models were developed. The models were evaluated using 50 Monte Carlo splits. TabICLv2 performed slightly better for MS prediction, with an RMSE of 1.49 ± 0.22 kN and an R^2^ of 0.85 ± 0.04, whereas TabPFN showed a slight advantage for ITS prediction, achieving an RMSE of 0.23 ± 0.08 MPa and an R^2^ of 0.91 ± 0.06. Furthermore, Pareto filtering identified nine non-dominated mixtures, and TOPSIS ranking selected the highest-ranked equal-weight compromise mixture, with MS = 15.23 kN and ITS = 3.90 MPa. The results indicate that mineral fibers are more suitable for improving the balanced performance of MS and ITS, carbon fibers are more favorable for improving MS, and plastic fibers are more effective in improving ITS. Finally, a Streamlit-based graphical user interface was developed to enable real-time prediction and MS–ITS trade-off visualization, providing a reference for preliminary mix design of asphalt concrete.

## 1. Introduction

Asphalt concrete serves as a fundamental material in a wide range of civil infrastructure applications, particularly in highway pavements [1], airport surfaces [2], parking facilities [3], and impermeable core structures in embankment dams [4]. Its popularity stems from a combination of favorable engineering characteristics, such as adequate strength, water resistance, and economic efficiency throughout its service life [5,6,7]. However, the performance of asphalt concrete gradually changes when exposed to repeated loading, temperature fluctuations, oxidative aging, and other environmental influences [8,9,10]. These interacting factors can progressively deteriorate the mechanical performance of asphalt concrete and reduce the long-term reliability of engineering structures. Consequently, developing effective approaches for assessing and forecasting the mechanical behavior of asphalt concrete has become an essential task for material design, quality control, and infrastructure management.

Among the commonly used mechanical performance indicators, Marshall Stability (MS) and Indirect Tensile Strength (ITS) are two important parameters for evaluating asphalt concrete. MS is generally used to characterize the stability and deformation resistance of asphalt mixtures under loading [11], while ITS reflects the tensile resistance and cracking-related performance [12]. The two indicators describe different aspects of mechanical behavior and are not always improved simultaneously. A mixture with high MS may not necessarily exhibit excellent tensile resistance, and a mixture with high ITS may not always have the best stability or deformation resistance. Therefore, focusing only on a single performance indicator may lead to incomplete or biased mixture evaluation. Simultaneous consideration of MS and ITS is more suitable for assessing the balanced mechanical performance of asphalt concrete and supporting multi-objective mixture selection.

Traditional experimental methods provide direct and reliable measurements of asphalt mixture performance, but they are usually time-consuming, labor-intensive, and costly [13,14,15,16,17,18]. Changes in asphalt properties, aggregate gradation, volumetric parameters, fiber characteristics, and production-related conditions often require repeated laboratory tests to evaluate their effects on MS and ITS. In addition, the mechanical behavior of asphalt concrete is governed by complex nonlinear interactions among multiple variables, such as asphalt content, air voids, gradation structure, and fiber content. Empirical equations and simple statistical methods are usually limited by predefined assumptions and specific data ranges, making it difficult to accurately capture these interactive effects [19,20]. Moreover, traditional experimental or empirical approaches provide limited support for rapidly identifying mixtures with balanced MS and ITS performance from existing experimental data [21,22].

Machine learning provides a promising alternative for modeling the nonlinear relationship between mixture design variables and mechanical properties. In recent years, data-driven methods have been increasingly applied to predicting the properties of concrete materials, including compressive strength, tensile strength, elastic modulus, durability-related indicators, and other engineering performance parameters [23,24,25]. Traditional machine learning models, such as Extreme Gradient Boosting (XGBoost) and Random Forest (RF), have also been widely employed in asphalt concrete research to predict key performance indicators such as Marshall stability, splitting strength, rutting resistance, and stiffness modulus [26,27].

Although previous studies have achieved encouraging results using machine learning techniques, the majority of adopted models are still based on conventional algorithms, such as neural-network models, bagging ensembles, and boosting ensembles. Their performance heavily depends on large dataset-specific training procedures and extensive hyperparameter tuning to achieve satisfactory predictive capability [28,29]. Recently, tabular foundation models, represented by Tabular Prior-data Fitted Network (TabPFN) and Tabular In-Context Learning version 2 (TabICLv2), have emerged as a new generation of machine learning methods for tabular data, showing promising potential in handling small-size, heterogeneous, and high-dimensional datasets with reduced tuning requirements [30,31]. In addition, existing studies have predominantly focused on predicting or optimizing a single performance indicator, whereas investigations that simultaneously consider multiple balanced mechanical properties, such as MS and ITS, are still limited.

To address the above issues, this study develops a machine-learning framework for the synthetic prediction of Marshall stability (MS) and indirect tensile strength (ITS) for the data-driven mixture selection. Specifically, three main innovations are as follows: (1) a heterogeneous database comprising 389 experimental asphalt mixtures and 15 features is established for the simultaneous prediction of MS and ITS, supporting the integrated assessment of deformation resistance and tensile resistance; (2) two tabular foundation models, TabPFN and TabICLv2, are systematically benchmarked against Optuna-tuned Random Forest and XGBoost models using 50 repeated Monte Carlo via 80/20 train-test splits, with target-specific prediction accuracy and stability reported separately for MS and ITS; and (3) an experimental data-driven mixture selection strategy combining Pareto filtering and TOPSIS ranking is implemented in the MS-ITS objective space to identify experimentally validated mixtures with balanced mechanical performance. The resulting prediction and trade-off analyses are further deployed in a Streamlit-based graphical user interface (GUI) for real-time prediction, visualization, and local interpretation.

## 2. Materials and Methods

**Figure 1:**
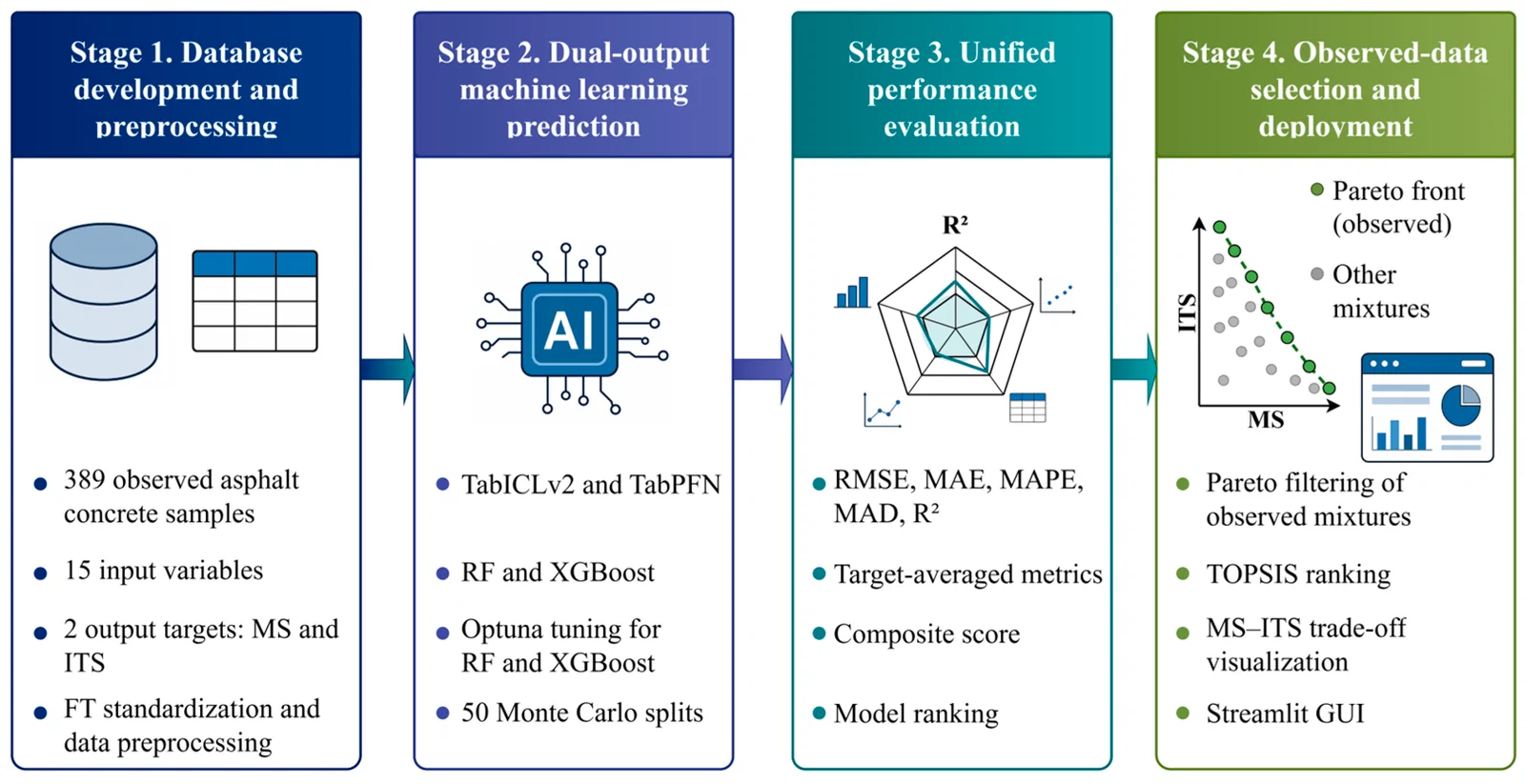
General research framework.

Figure 1 presents the general research framework of this study, which consists of four main stages: (1) An asphalt concrete database was constructed and preprocessed. The database contains 389 experimental samples, with 15 input variables describing asphalt properties, aggregate gradation, volumetric parameters, and fiber characteristics. MS and ITS were regarded as two output targets. (2) Dual-output machine learning models were developed to predict MS and ITS simultaneously. Two tabular models, TabICLv2 and TabPFN, were compared with two traditional tree-based ensemble models, RF and XGBoost. For RF and XGBoost, Optuna was further used for hyperparameter tuning. (3) Model performance was evaluated through Monte Carlo cross-validation using multiple indicators, including RMSE, MAE, MAPE, MAD, and R^2^. These metrics were further integrated into a composite score to support unified model comparison and ranking. (4) The experimentally observed mixtures were screened using Pareto filtering in the MS–ITS objective space, and the retained Pareto samples were ranked by TOPSIS. The selected models and trade-off analysis results were then integrated into a Streamlit-based GUI to support performance prediction, visualization, and preliminary mixture selection.

### 2.1. Dataset Preparation and Preprocessing

#### 2.1.1. Data Collection and Variable Description

A dataset of 389 asphalt concrete mixtures was collected from 42 published studies covering the period 2003–2024, providing the basis for synthetic modeling MS and ITS [32,33,34,35,36,37,38,39,40,41,42,43,44,45,46,47,48,49,50,51,52,53,54,55,56,57,58,59,60,61,62,63,64,65,66,67,68,69,70,71,72,73]. To represent the main mixture design factors, 15 input variables were selected and grouped according to asphalt binder properties, aggregate gradation, volumetric indices, and fiber characteristics [26]. The asphalt-related features included penetration (Pe), ductility (Du), softening point (SP), and asphalt content (AC). The aggregate- and volumetric-related features included the sieve passing percentages of 2.36 mm, 4.75 mm, and 9.5 mm aggregates (Ag2.36, Ag4.75, and Ag9.5); air voids (AVs); voids in mineral aggregate (VMAs); and voids filled with asphalt (VFAs). The fiber-related features included fiber type (FT), fiber content (FC), fiber length (FL), fiber tensile strength (TS), and fiber melting temperature (MT). In contrast to previous single-output studies that focused on only one mechanical property, both MS and ITS were used as output variables in this study to support multi-target performance prediction and subsequent multi-objective mixture design optimization.

For the categorical feature FT, the original fiber names were standardized into seven representative groups, including plastic fiber, mineral fiber, bio-fiber, carbon fiber, glass fiber, steel fiber, and no fiber. Among the 389 samples, 237 samples contained fiber reinforcement, whereas 152 samples were non-fiber asphalt concrete mixtures. The distribution of fiber categories is shown in Table 1.

#### 2.1.2. Data Analysis

Table 2 reports the descriptive statistics for all input and output variables, including extreme values, quartiles, means, and standard deviations. Overall, the dataset spans broad ranges of binder properties, gradation indices, volumetric parameters, fiber descriptors, and production conditions, reflecting the diversity of the collected experimental sources. Asphalt-related variables, such as penetration, ductility, softening point, and asphalt content, all show varying degrees of variation, reflecting differences in asphalt materials and mixture designs. The aggregate gradation variables Ag2.36, Ag4.75, and Ag9.5 also have wide value ranges, indicating that the database contains asphalt mixtures with different gradations. Fiber-related variables, particularly for fiber length, tensile strength, melting temperature, and fiber content, also showed substantial dispersion, because the database included different fiber types as well as non-fiber mixtures. Among two output variables, MS ranges from 3.40 to 26.72 kN, while ITS ranges from 0.20 to 3.94 MPa, indicating that the database covers asphalt concrete samples with different mechanical performances.

**Figure 2:**
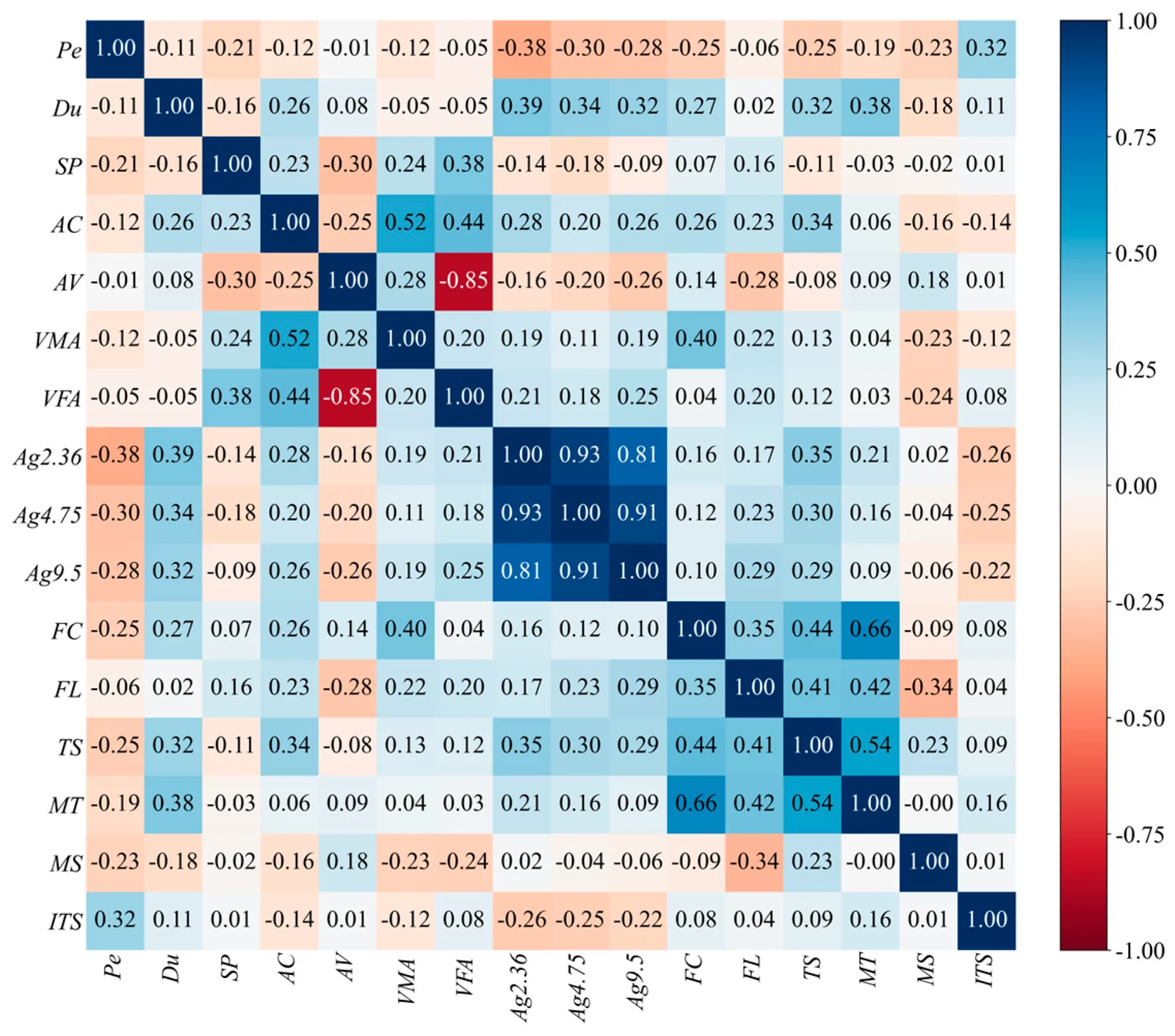
Correlation analysis of input and output variables.

Figure 2 gives the Pearson correlation matrix for the input descriptors and target responses. It is observed that high correlation coefficients appear mainly between variables with clear physical relationships. For example, different sieve passing indicators, including Ag2.36, Ag4.75, and Ag9.5, show strong positive correlations, because these variables synthetically characterize the gradation composition of the mixture. Meanwhile, relatively strong correlations are also observed among volumetric parameters such as AV, VMA, and VFA, which are consistent with their inherent relationships in definition and calculation. After excluding these expected physical associations, the absolute Pearson coefficients of all remaining feature pairs are below 0.7 [24], suggesting that no severe multicollinearity exists. Therefore, the selected variables provide complementary descriptions of mixture composition and mechanical behavior and were retained as inputs for subsequent machine learning analysis.

#### 2.1.3. Data Preprocessing

Before model training, the constructed database was preprocessed to ensure data format consistency and meet the input requirements of machine learning models [74]. For fiber-related variables, it was necessary to distinguish differences between “non-fiber samples” and “missing data”. For non-fiber samples, the values of FC, FL, TS, and MT were assigned 0 to indicate the absence of fiber and corresponding properties. Meanwhile, the categorical variable FT was unified into seven representative categories, including plastic fiber, mineral fiber, bio-fiber, carbon fiber, glass fiber, steel fiber, and no fiber. The final dataset consisted of 15 input variables and 2 output variables. To evaluate the prediction capability of models on unknown samples, the database was divided into training and testing sets at a ratio of 8:2 in each Monte Carlo random split. This random splitting process was repeated 50 times to reduce the influence of a single data partition on model evaluation results.

### 2.2. Machine Learning Models

Table 3 lists the four models evaluated in this work, including two tabular foundation models (TabICLv2 and TabPFN) and two conventional tree-based ensemble models (RF and XGBoost). The purpose of selecting these four types of models is to compare the prediction capability of emerging tabular foundation models and traditional machine learning models on a small-scale, multi-variable, and dual-output asphalt concrete dataset.

**Figure 3:**
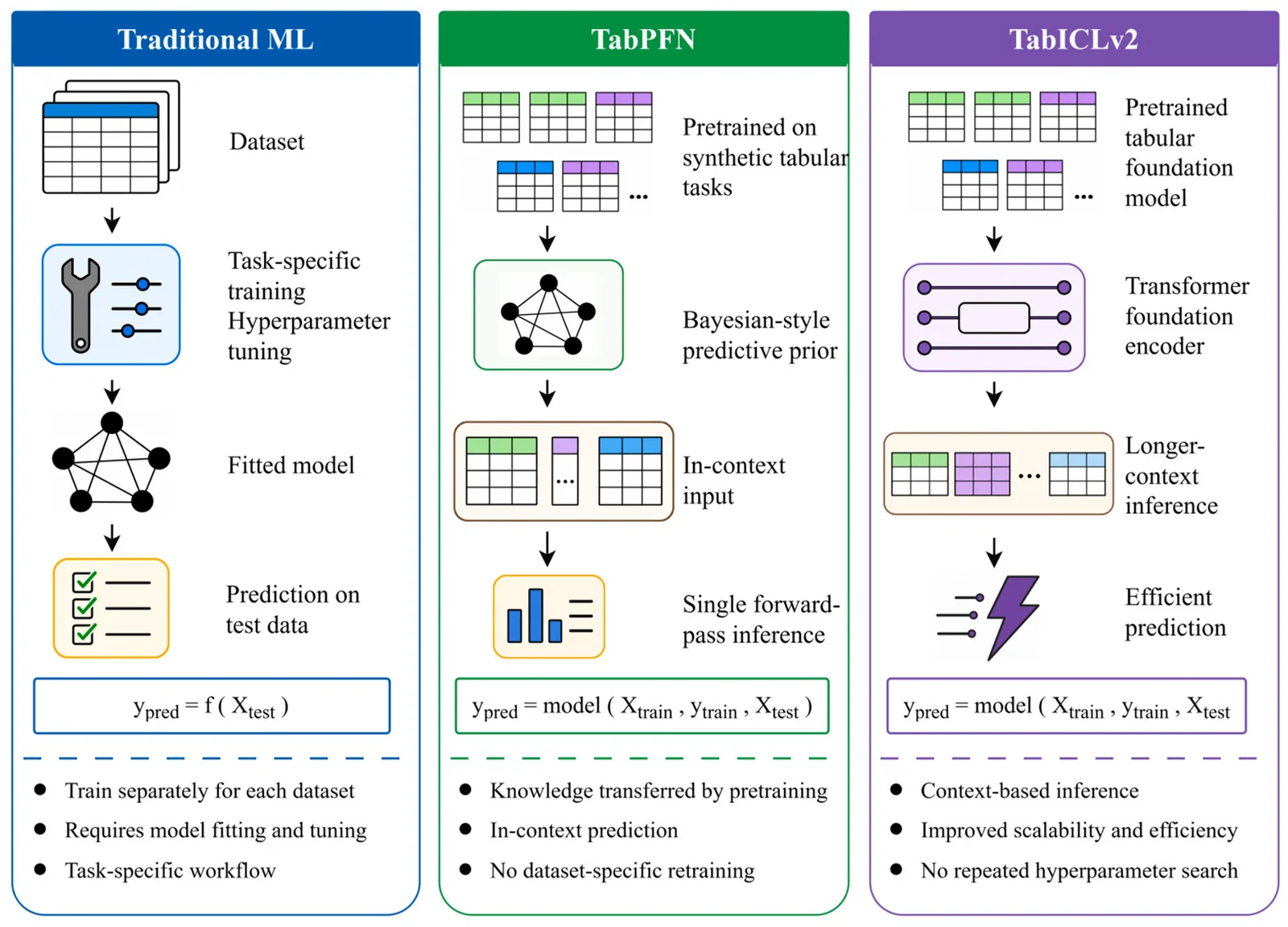
Comparison of traditional machine learning and tabular foundation models.

Figure 3 further illustrates the differences between traditional machine learning models and tabular foundation models. Conventional models are generally fitted directly on the task-specific dataset and learn relationships between mixture descriptors and target properties from those samples. For example, RF improves stability by aggregating the outputs of multiple decision trees [75], while XGBoost continuously corrects the prediction errors of the previous model through a stepwise boosting strategy [76], thereby improving model fitting ability and model generalization. These models have relatively good interpretability and a mature application basis, and are therefore suitable as traditional benchmark models in this study. Different from traditional models, TabPFN and TabICLv2 are recently developed tabular foundation models, whose core advantage lies in using pretraining or in-context learning mechanisms to handle tabular prediction tasks. TabPFN acquires general-purpose tabular modeling ability from extensive pretraining on synthetic tasks and uses contextual information to generate predictions for new small-sample regression tasks [30]. TabICLv2 is also based on the idea of pretrained tabular models and can extract potential relationships among variables from limited samples and directly apply them to the current prediction task [31]. Therefore, these models have potential advantages in material performance prediction with limited sample sizes and complex variable types.

### 2.3. Evaluation Metrics and Monte Carlo Validation

The prediction task in this study contains two response variables with different physical meanings and numerical scales: MS and ITS. For this reason, prediction errors were not pooled between wo outputs. Instead, each metric was computed separately for MS and ITS within each Monte Carlo split. The target-specific metrics were retained separately for performance reporting and were combined only after direction unification and dimensionless normalization in the composite evaluation.

Let *s* denote the index of a Monte Carlo split; *k* denote the target variable, where *k* belongs to {MS, ITS}; and *i* denote the sample index in the corresponding training or testing subset. For split *s*, the notation in Equation (1) represents the relationship of measured value, predicted value, and residual of target *k* for sample *i*:

(1) ei,k(s)=yi,k(s)−y^i,k(s)

where s is the Monte Carlo split index; k∈{MS,ITS} identifies the predicted target; i is the sample index; and $y_{i,k}^{\left(s\right)}$, y^i,k(s), and $e_{i,k}^{\left(s\right)}$ denote the measured value, predicted value, and residual of target k for sample i in split s, respectively.

Based on the target-specific residuals, five indicators were calculated for each target. The root mean square error (RMSE) and mean absolute error (MAE) quantify the magnitude of prediction errors in the original unit of the target variable, whereas the mean absolute percentage error (MAPE) expresses the relative error as a percentage. The coefficient of determination (R^2^) measures the explained variance, and the median absolute deviation (MAD) was calculated from the residuals around their median to provide a robust description of residual dispersion.

(2) RMSEk(s)=1ns∑i=1ns(yi,k(s)−y^i,k(s))2

(3) MAPEk(s)=100ns∑i=1nsyi,k(s)−y^i,k(s)yi,k(s)

(4) MAEk(s)=1ns∑i=1nsyi,k(s)−y^i,k(s)

(5) Rk2,(s)=1−∑i=1ns(yi,k(s)−y^i,k(s))2∑i=1ns(yi,k(s)−y¯k(s))2

(6) $${\mathrm{MAD}}_{k}^{\left(s\right)}={\mathrm{median}}_{i}\left|e_{i,k}^{\left(s\right)}-{\mathrm{median}}_{j}(e_{j,k}^{\left(s\right)})\right|$$

In Equations (2)–(6), $n_{s}$ denotes the number of samples in the evaluated subset of split s; ${\bar{y}}_{k}^{\left(s\right)}$ is the mean measured value of target k in that subset; and ${\mathrm{median}}_{i}$ and ${\mathrm{median}}_{j}$ indicate median operations over the corresponding sample indices. Smaller RMSE, MAPE, MAE, and MAD values are related to lower prediction errors, while a larger $R^{2}$ indicates stronger agreement between measured and predicted values.

The metric results for MS and ITS were retained as a two-target vector for every split:

(7) $$M^{s}=\left(M_{MS}^{s},M_{ITS}^{s}\right),\;k\in \left\{MS,ITS\right\}$$

To reduce sensitivity to a particular random data partition, the complete training and testing procedure was repeated over 50 Monte Carlo splits. For each metric M and each target k, the mean test-set performance and its standard deviation were calculated separately as follows:

(8) $${\bar{M}}_{k}=\frac{1}{S}\Sigma_{s=1}^{S}M_{k}^{s}$$

(9) $$SD\left(M_{k}\right)=\sqrt{\frac{1}{S-1}\Sigma_{s=1}^{S}{\left(M_{k}^{s}-{\bar{M}}_{k}\right)}^{2}}$$

In Equations (7)–(9), M represents any one of the five evaluation metrics; $M_{k}^{s}$ denotes the value of metric M for target k in Monte Carlo split s; $M^{s}$ is the two-target metric vector; ${\bar{M}}_{k}$ is the mean of the target-specific metric values across S splits (*S* = 50); and $SD\left(M_{k}\right)$ is the corresponding standard deviation.

Therefore, all reported RMSE, MAPE, MAE, R^2^, and MAD values remain target specific. No raw MS and ITS metric values were averaged in the performance summary, due to their different units and numerical scales.

### 2.4. Model Ranking and Comprehensive Evaluation

The five metrics describe complementary aspects of model performance and have different optimization directions. To avoid combining MS and ITS errors before considering their different units and numerical scales, all metrics were processed separately for each target. The composite score was calculated only from direction-consistent, dimensionless normalized scores and was used exclusively for relative model ranking.

For each candidate model *m*, metric *q* ∈ {RMSE, MAPE, MAE, R^2^, MAD}, and target *k* ∈ {MS, ITS}, the target-specific test metric $x_{m,q,k}$ was first transformed into a direction-consistent utility value. Smaller values are preferred for RMSE, MAPE, MAE, and MAD, whereas a larger $R^{2}$ is preferred:

(10) $$\begin{array}{l} u_{m,q,k}=-x_{m,q,k}\;for\;q\in \left\{RMSE,MAPE,MAE,MAD\right\};\;\\ u_{m,q,k}=x_{m,q,k}\;for\;q=R^{2} \end{array}$$

For each target-metric axis, the utility values were standardized among all candidate models. The mean $\mu_{q,k}$ and standard deviation $\sigma_{q,k}$, computed over all candidate models m, were therefore calculated separately for every combination of metric *q* and target *k*:

(11) $$z_{m,q,k}=\frac{u_{m,q,k}-\mu_{q,k}}{\sigma_{q,k}}$$

The standardized values were then mapped to a bounded interval using a sigmoid transformation with a fixed scaling parameter, ensuring that the normalized scores remained within [0, 1] while preserving the relative ordering of candidate models:

(12) $$S_{m,q,k}=\frac{1}{1+\exp\left(-\frac{z_{m,q,k}}{\alpha }\right)}$$

The final dimensionless composite score for each model m was obtained as the equal-weight mean of all ten normalized target-metric scores (2 targets × 5 metrics = 10 axes):

(13) $$Composite_{m}=\frac{1}{10}\Sigma_{k\in \left\{MS,ITS\right\}}\Sigma_{q=1}^{5}S_{m,q,k}$$

For the five-axis radar chart and stacked-bar display in Section 3.2, the separately normalized MS and ITS scores were averaged within each metric to produce a visualization-only score ${\bar{S}}_{m,q}$ that does not enter the official composite calculation:

(14) $${\bar{S}}_{m,q}=\frac{S_{m,q,MS}+S_{m,q,ITS}}{2}$$

This visualization-level aggregation is performed after target-specific normalization and does not represent a direct average of raw RMSE, MAE, MAPE, MAD, or R^2^ values. A larger composite score indicates a more balanced overall performance among prediction accuracy, relative error, explained variance, and residual robustness.

### 2.5. Hyperparameter Tuning Through Optuna

To improve the prediction performance of traditional tree-based ensemble models, Optuna was used in this study to automatically optimize the key hyperparameters of RF and XGBoost [77]. On contrast, TabICLv2 and TabPFN are tabular foundation models whose predictive ability is mainly derived from pretraining or in-context learning. Therefore, their default configurations were adopted in this study, and no additional hyperparameter search was performed for TabICLv2 and TabPFN. The computational environment and versions of the key Python packages used for model development, statistical analysis, visualization, and GUI deployment are summarized in Table A1.

**Figure 4:**
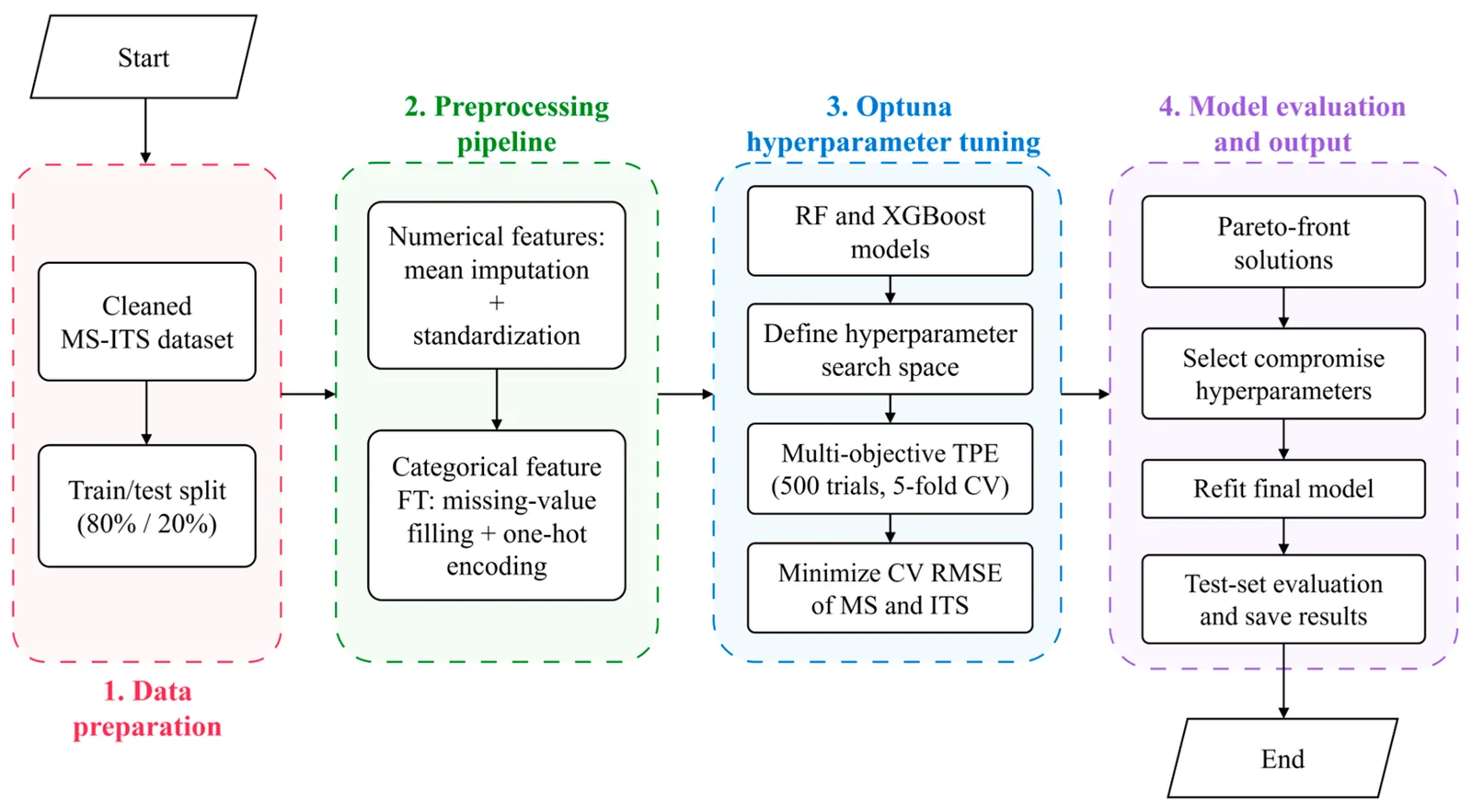
Workflow of Optuna-based hyperparameter tuning for tree-based models.

Figure 4 shows the Optuna-based hyperparameter tuning workflow. First, the corresponding hyperparameter search spaces were defined according to the characteristics of RF and XGBoost. Then, in each trial, Optuna automatically generated a candidate set of hyperparameters and constructed the corresponding model on the training set. After model training, the objective function values were calculated through 5-fold cross-validation within the training set, where the cross-validation process was randomly shuffled and the random seed was set to 42. For each model to be optimized, Optuna performed 500 trials to search for a better hyperparameter combination within the predefined search space. Since this study simultaneously predicted two mechanical performance indicators, MS and ITS, a multi-objective optimization strategy was adopted. Specifically, the cross-validation RMSE values of MS and ITS were used as two independent optimization objectives, and both values were minimized simultaneously through the multi-objective optimization mechanism of Optuna. After optimization, the compromise solution with the smallest normalized average error was selected from the Pareto-optimal solution set as the final hyperparameter combination. After the hyperparameter search was completed, the selected hyperparameter combination was used for subsequent model training and testing evaluation. Through this tuning process, the prediction stability and generalization capability of traditional tree-based ensemble models in the MS–ITS dual-output prediction task can be improved while maintaining a fair model comparison.

### 2.6. Pareto Filtering and TOPSIS-Based Selection of Observed Experimental Mixtures

**Figure 5:**
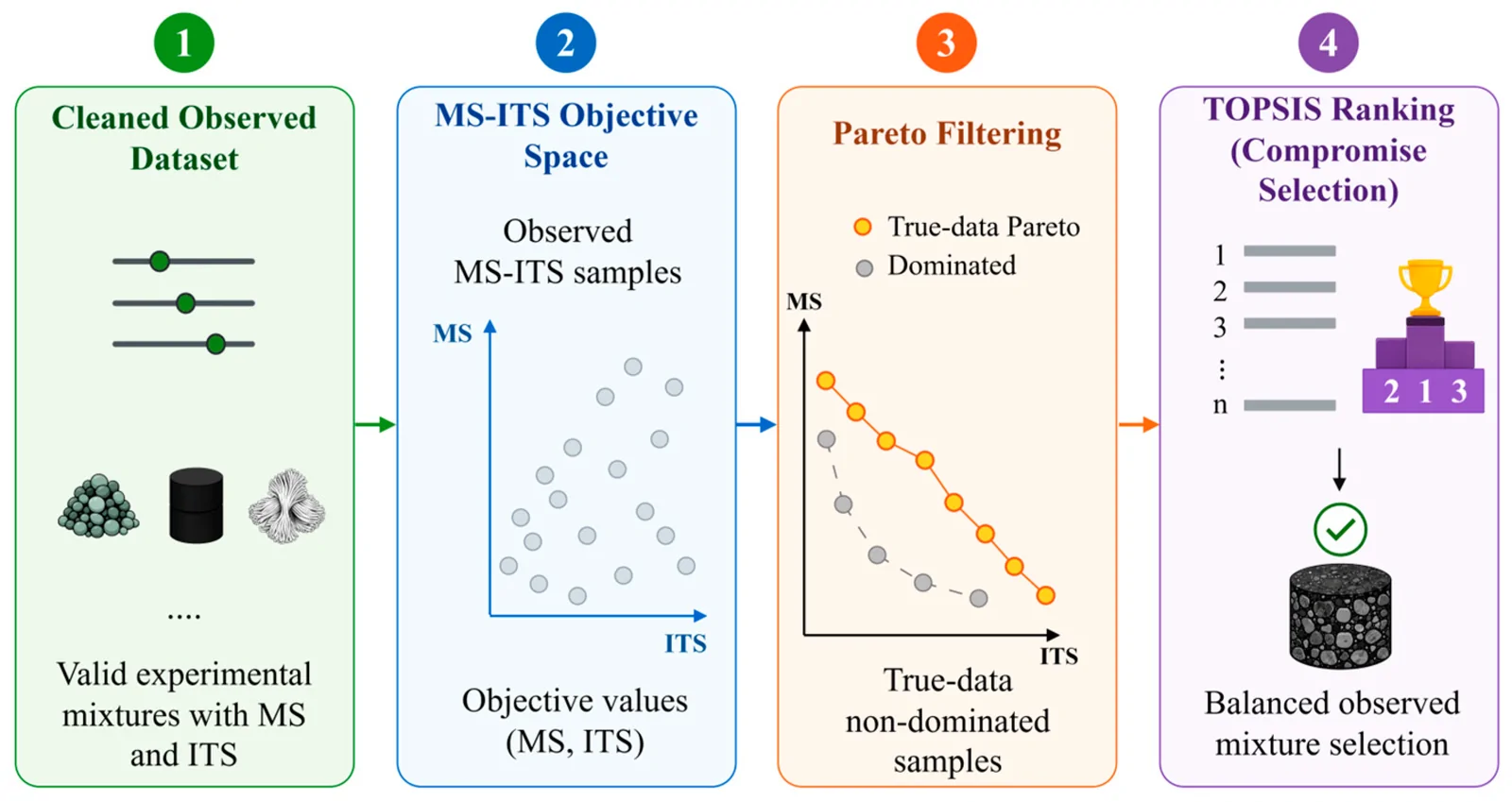
Workflow of observed-data Pareto filtering and TOPSIS-based mixture selection.

To support multi-objective mixture selection using only experimental data, the cleaned database was filtered first to retain samples with valid MS and ITS values. Both MS and ITS were treated as benefit criteria because larger values indicate better mechanical performance. As shown in Figure 5, the observed-data selection workflow consisted of three main steps: valid-sample filtering, Pareto-based non-dominated mixture identification, and TOPSIS-based ranking of the retained Pareto samples.

Specifically, non-dominated observed mixtures were identified by Pareto filtering in the two-dimensional MS–ITS objective space [78]. A mixture was retained as a true-data Pareto sample when no other observed mixture simultaneously achieved equal or higher MS and ITS with at least one strictly higher objective value. The retained Pareto candidates were subsequently prioritized with TOPSIS [79,80]. The equal-weight scenario, in which MS and ITS were assigned weights of 0.5 and 0.5, respectively, was used as the primary ranking scheme.

## 3. Results and Discussion

### 3.1. Hyperparameter Optimization Results

**Figure 6:**
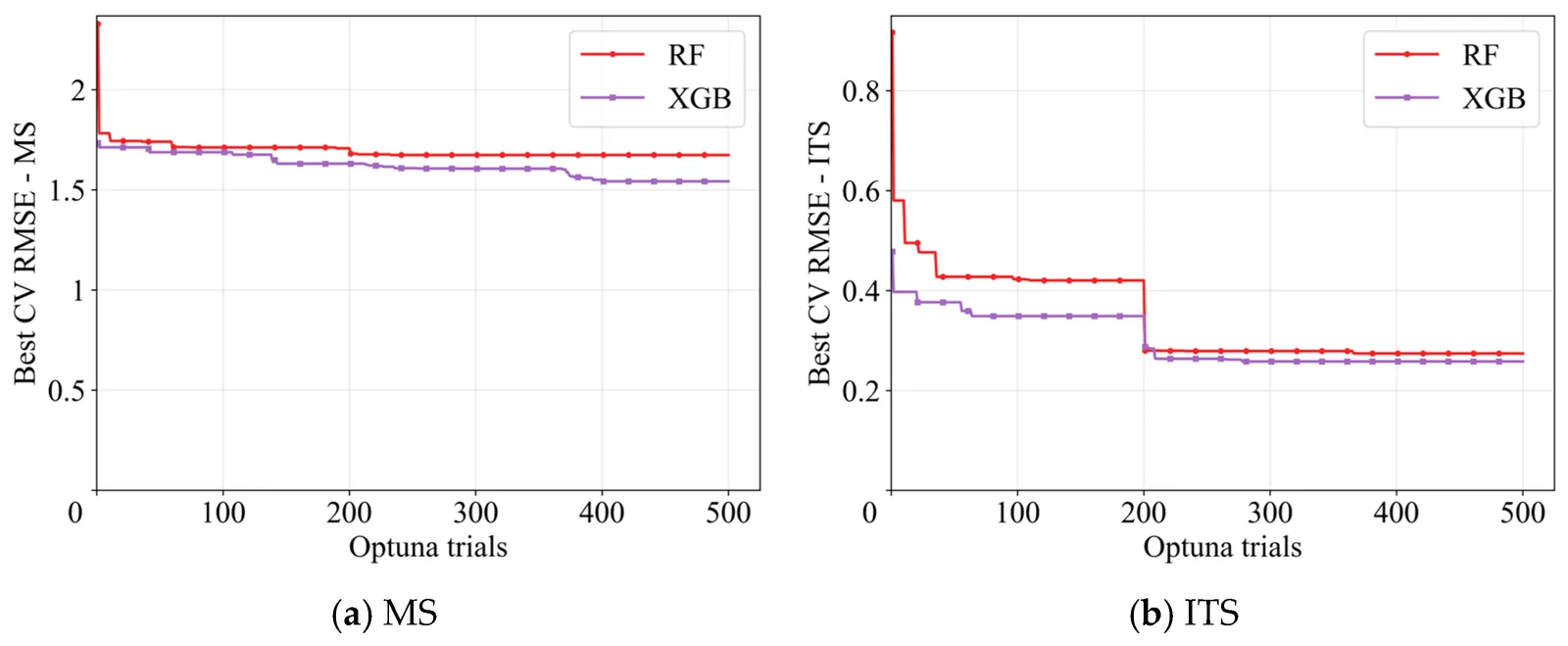
Optimization histories of RF and XGBoost during Optuna tuning.

Figure 6 shows the optimization histories of RF and XGBoost during Optuna tuning. In this study, the cross-validation RMSE values of MS and ITS were used as two independent optimization objectives, and the compromise solution with the smallest normalized average error was selected from the Pareto-optimal solution set as the final hyperparameter combination. The predefined hyperparameter distributions and search ranges for RF and XGBoost are summarized in Table A2 in Appendix B. From the optimization process, the objective function values of both RF and XGBoost decreased noticeably in the early trials, indicating that Optuna could gradually eliminate poor parameter combinations within the predefined search space and locate parameter regions more suitable for the MS–ITS dual-output prediction task. As the number of trials increased, further improvement in the best cross-validation error gradually became smaller, and the optimization curves generally tended to converge, suggesting that 500 trials were sufficient for hyperparameter search of traditional tree-based models in this study. It should be noted that, because MS and ITS were optimized simultaneously, the final parameter combination did not simply pursue the lowest error for a single objective, but reflected a compromise optimization result between the two mechanical performance indicators.

According to the optimization results, RF finally adopted a relatively large number of trees and a deep tree structure to enhance its ability to fit nonlinear relationships among variables. XGBoost adopted relatively large number of weak learners, shallow tree depth, and moderate regularization parameters to balance model complexity and generalization capability. The specific hyperparameter settings are shown in Table 4. Based on the optimized parameter combinations, RF and XGBoost were further included in the unified Monte Carlo testing evaluation together with TabICLv2 and TabPFN to compare their generalization performance in the MS and ITS dual-output prediction task.

### 3.2. Prediction Performance

**Figure 7:**
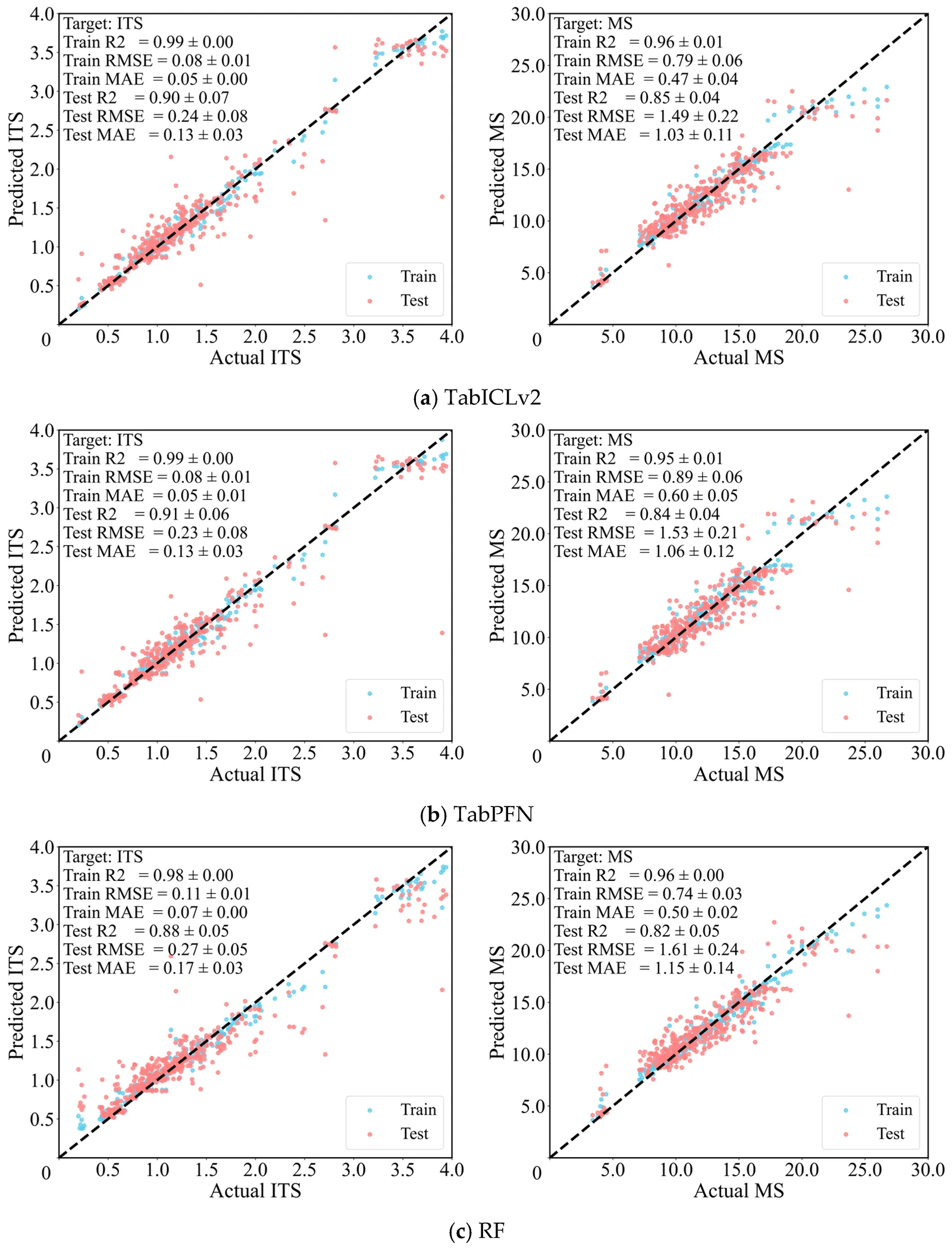

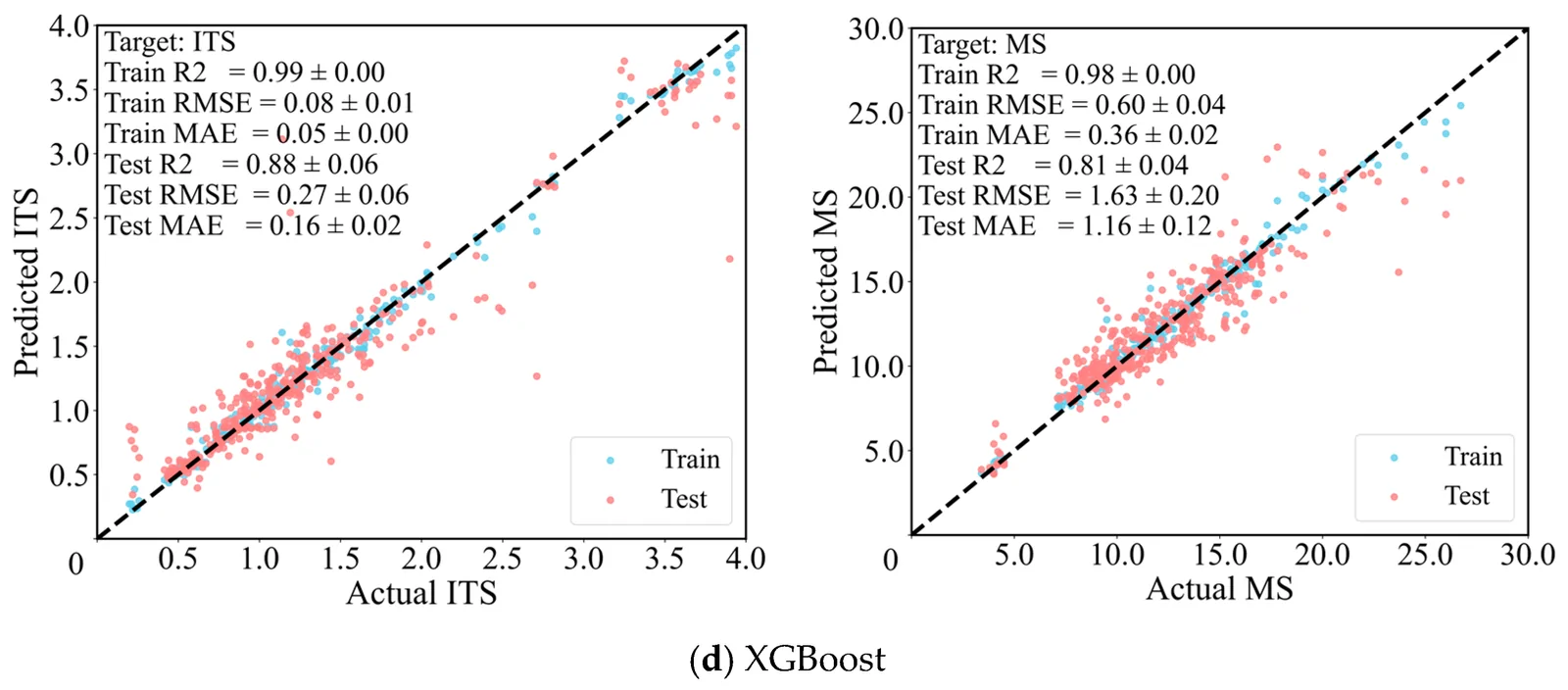
Measured and predicted values of MS and ITS for different models.

Figure 7 compares the measured and predicted values of the four models across 50 Monte Carlo train–test splits. Overall, the prediction points of TabICLv2, TabPFN, RF, and XGBoost are generally distributed around the reference line, indicating that all four models are able to capture the nonlinear relationships between the input variables and the MS and ITS responses. Among the evaluated models, the prediction points of TabICLv2 and TabPFN are more distributed around the reference line, especially for the ITS task, indicating better agreement between the measured and predicted values. On contrast, RF and XGBoost exhibit relatively greater dispersion for several high and low performance samples.

Table 5 further presents the specific test-set values of all evaluation metrics for four models across 50 Monte Carlo splits. For MS prediction, TabICLv2 performed slightly better performance than TabPFN, achieving a lower RMSE (1.49 ± 0.22 vs. 1.53 ± 0.21 kN) and a higher R^2^ (0.85 ± 0.04 vs. 0.84 ± 0.04). For ITS prediction, TabPFN showed a slight advantage, with lower RMSE and MAPE and higher R^2^. RF and XGBoost generally produced larger prediction errors than two tabular foundation models. Overall, TabICLv2 showed clearer advantage in MS prediction, whereas TabPFN performed slightly better performance in ITS prediction; both models demonstrated strong potential for small-sample, multivariable, and dual-output prediction tasks. In addition, Table 5 reports the computational speeds of all models on an Intel Core i7-12800HX CPU and an NVIDIA GeForce RTX 4070 Laptop GPU (8 GB), expressed as the average runtime per Monte Carlo split.

**Figure 8:**
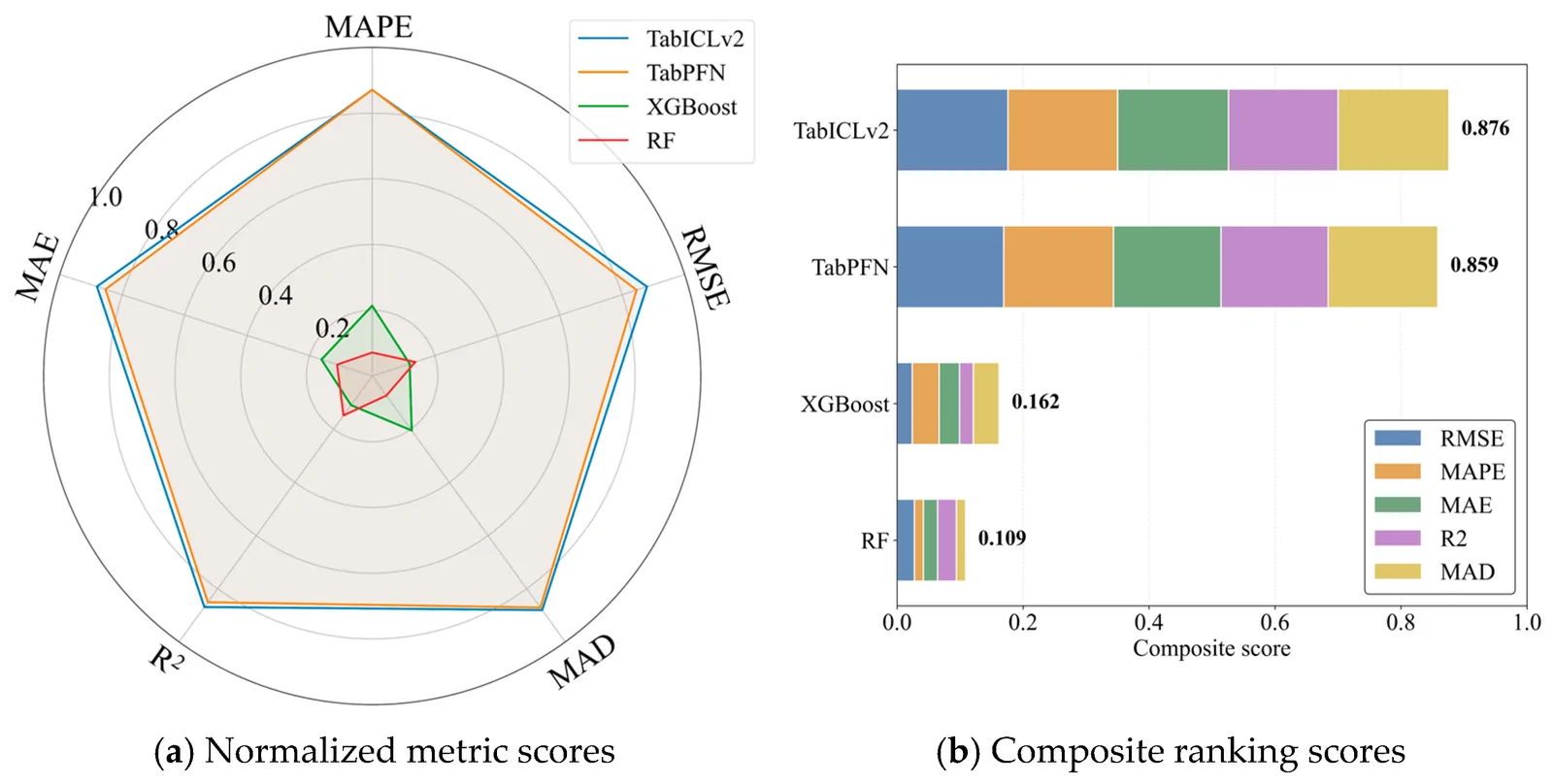
Comprehensive comparison of model performance based on multiple metrics.

To provide a unified comparison of model performance, Figure 8 presents a comprehensive evaluation based on the direction-consistent and dimensionless scores of five metrics. Figure 8a shows the normalized scores for five evaluation metrics. Overall, TabICLv2 and TabPFN achieved higher scores than RF and XGBoost among most metric dimensions, indicating that two tabular models provided more balanced prediction performance. Their score profiles were similar, although TabPFN showed a slight advantage in MAPE, whereas TabICLv2 performed slightly better on the other four metrics. Figure 8b further integrates five normalized metrics into an equal-weight composite score for overall model ranking. TabICLv2 achieved the highest composite score 0.876, followed closely by TabPFN with a score of 0.859. In comparison, RF and XGBoost performed much worse than two tabular models in comprehensive evaluation metrics, but they still maintained acceptable prediction accuracy and can therefore serve as reliable benchmark models.

To determine whether the observed performance differences were statistically significant, formal paired comparisons were conducted across 50 matched Monte Carlo splits. As summarized in Table A3 in Appendix B, the Friedman tests indicated significant overall differences among four models for all evaluation metrics of both MS and ITS. Subsequent pairwise Wilcoxon signed-rank tests with Holm correction further showed that TabPFN and TabICLv2 significantly outperformed RF and XGBoost in all reported comparisons. Notably, TabPFN and TabICLv2 achieved these competitive results using default configurations, whereas RF and XGBoost underwent 500 Optuna tuning trials, indicating strong performance with substantially less task-specific hyperparameter tuning.

**Figure 9:**
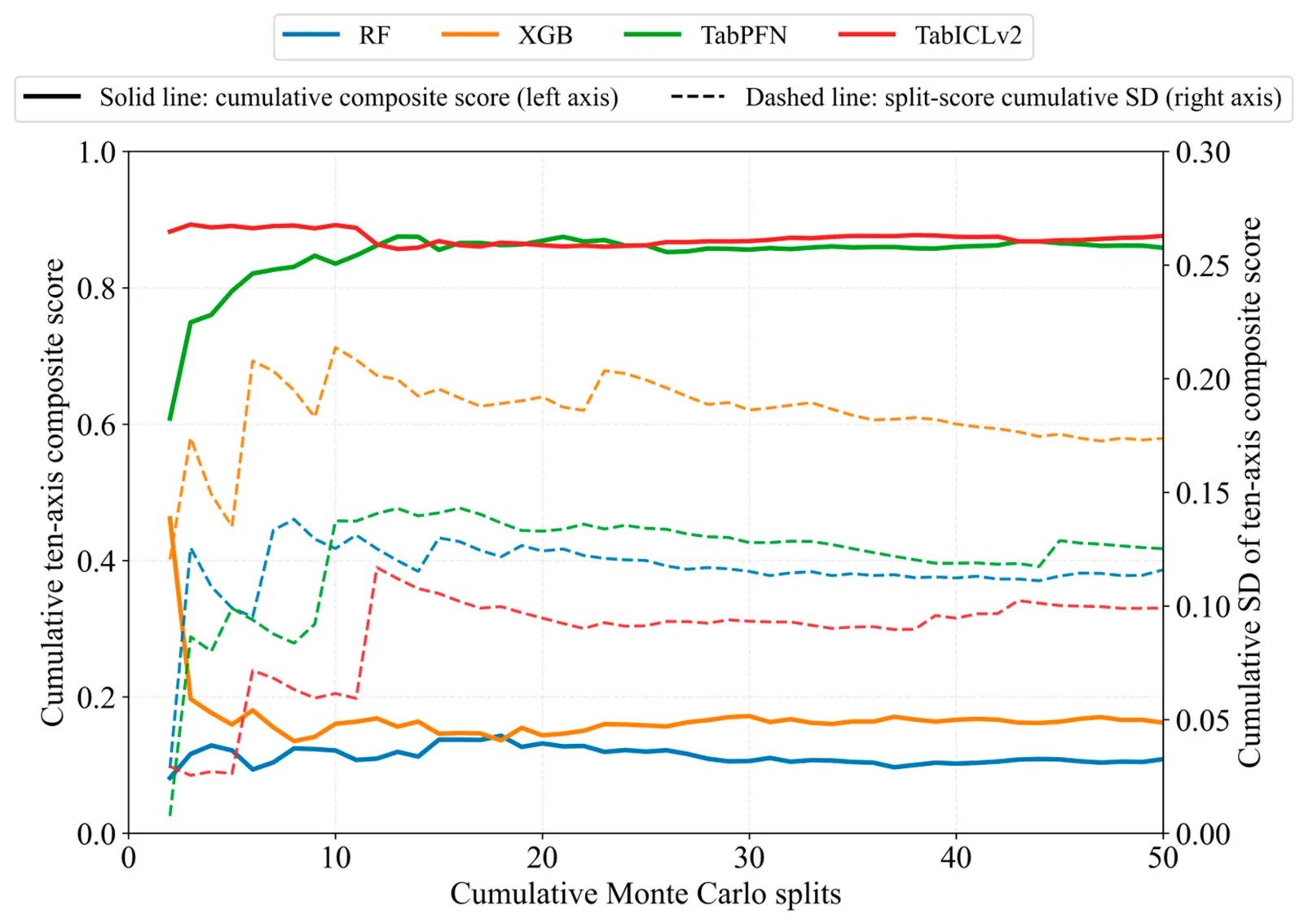
Stability of model composite-score estimates across 50 Monte Carlo train-test splits.

Figure 9 further demonstrates the robustness of the model evaluation across 50 Monte Carlo train–test splits. Although the cumulative mean composite scores fluctuated during the early splits, they gradually stabilized as the number of repetitions increased. TabICLv2 and TabPFN consistently maintained higher cumulative mean scores than RF and XGBoost, indicating that the relative model ranking was not affected by individual data partitions. The cumulative standard deviations, shown by the dashed lines, also became increasingly stable in the later splits, supporting the reliability of the performance estimates obtained from the full 50-split evaluation.

### 3.3. Sensitivity Analysis of Non-Fiber Encoding

**Figure 10:**
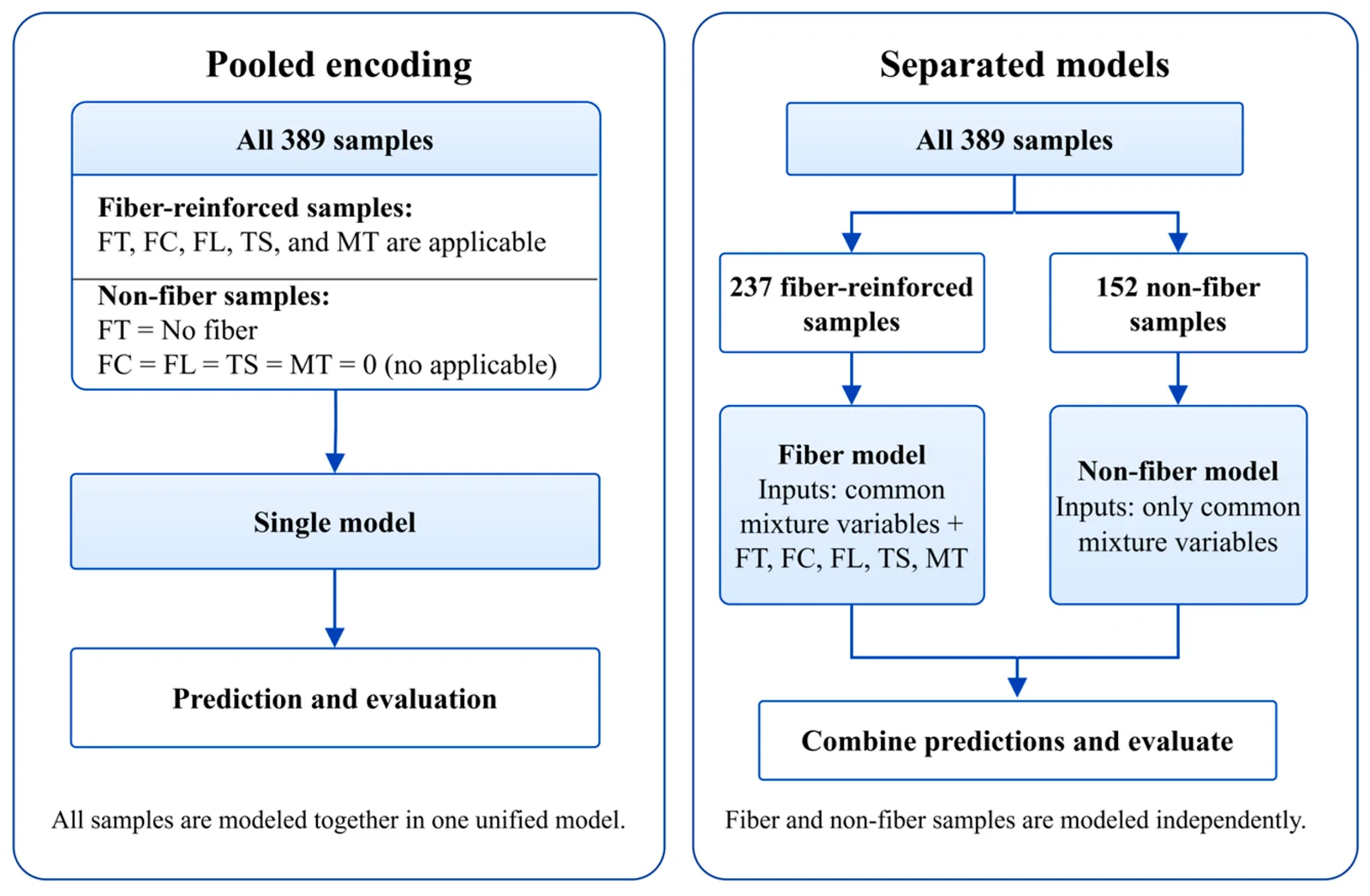
Pooled and separated strategies for non-fiber samples.

To evaluate whether the encoding of non-fiber samples affects prediction results, Figure 10 illustrates two comparable modeling strategies. Under pooled encoding, all 389 samples were included in a single model. For 237 fiber-reinforced samples, FT, FC, FL, TS, and MT were used as applicable fiber-related descriptors. For 152 non-fiber samples, FT was coded as No_fiber, whereas FC, FL, TS, and MT were set to be 0 because these variables were not applicable. Under the separated-model strategy, fiber-reinforced and non-fiber samples were modeled independently. The fiber model used both common mixture variables and fiber-related variables, whereas the non-fiber model used only 10 common mixture variables applicable to both groups. The predictions from the two subgroup models were then combined for evaluation. This design enables a direct comparison between pooled encoding and fiber-status-specific modeling under matched data partitions.

Table 6 further quantifies the performance of the two strategies using TabPFN across 50 matched Monte Carlo splits. For all test samples, pooled encoding achieved higher R^2^ and lower RMSE values for both MS and ITS. For MS, the pooled encoding strategy obtained an R^2^ of 0.837 ± 0.043 and an RMSE of 1.529 ± 0.209 kN, compared with 0.795 ± 0.062 and 1.710 ± 0.268 kN for the separated models. For ITS, the corresponding values were 0.903 ± 0.069 and 0.231 ± 0.078 MPa for pooled encoding, versus 0.877 ± 0.083 and 0.261 ± 0.080 MPa for the separated models. The paired Wilcoxon tests showed significant RMSE differences for all samples and the non-fiber subset for both targets. For the fiber-only subset, the difference was significant for MS (*p* = 0.004) but not for ITS (*p* = 0.126). These results indicate that pooled encoding generally provided better predictive performance, with the clearest improvements observed for non-fiber samples, while maintaining comparable ITS performance for fiber-reinforced samples.

### 3.4. Observed-Data Pareto Front and TOPSIS-Based Mixture Selection

#### 3.4.1. Observed Pareto Front and TOPSIS Ranking

**Figure 11:**
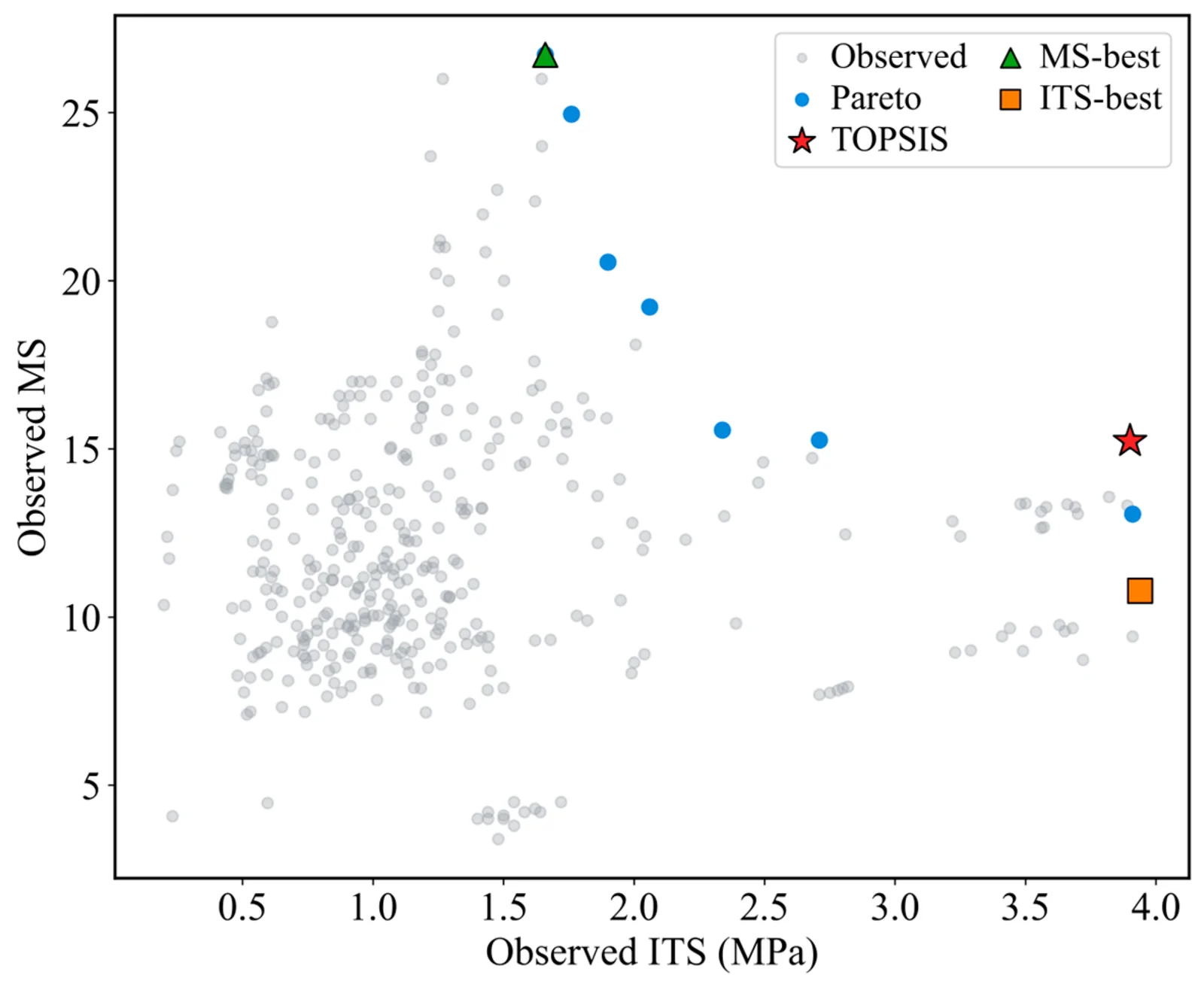
Pareto front and TOPSIS selection for observed MS-ITS data.

The observed-data selection results are summarized in Figure 11. After filtering the experimental database, 389 samples with valid MS and ITS values were retained for multi-objective evaluation, among which only 9 samples were identified as Pareto samples. These samples represent the experimentally observed upper boundary of the MS-ITS objective space, where no other observed mixture simultaneously achieved equal-or-higher MS and ITS with at least one higher objective value.

Table 7 presents the complete feature information and equal-weight TOPSIS ranking results of 9 Pareto samples. E122 ranked first, with MS = 15.23 kN, ITS = 3.90 MPa, and a TOPSIS score of 0.572, followed by E112 with a TOPSIS score of 0.522. Although E122 did not achieve the maximum value of either individual objective, it combined a high-level ITS value with a moderate-to-high MS value and therefore represented the best compromise solution under the equal-weight scenario. In contrast, E263 achieved the highest MS of 26.72 kN but a relatively low ITS of 1.66 MPa, whereas E239 achieved the highest ITS of 3.94 MPa but a lower MS of 10.78 kN, further illustrating the trade-off between the two mechanical properties.

#### 3.4.2. Design Implications for Mixture Selection

Table 8 summarizes the main design pathways derived from the Pareto samples. For the balanced MS–ITS pathway, E122 and E112 both employed mineral-fiber systems, with fiber contents of 0.20–0.30%, a fiber length of 6 mm, and asphalt contents of 5.27–5.80%. Their MS values ranged from 13.06 to 15.23 kN, while their ITS values remained high within 3.90–3.91 MPa. These results suggest that, within the current database, low-dosage mineral-fiber systems with a fiber length of 6 mm may provide a candidate design window for balanced stability and tensile resistance.

For the MS-dominant pathway, the carbon-fiber samples E263, E265, E266, and E267 achieved higher MS values of 19.22–26.72 kN, with fiber contents of 0.40–0.80%, a fiber length of 6 mm, and asphalt contents of 5.90–6.70%; however, their ITS values were limited to 1.66–2.06 MPa. On contrast, the ITS-dominant sample E239, which used plastic fiber at a content of 0.23%, achieved the highest ITS of 3.94 MPa but a comparatively lower MS of 10.78 kN. In addition, the no-fiber sample E069 remained on the Pareto front, indicating that fiber reinforcement is not the only route to obtaining a favorable MS–ITS combination, while E126 provides an additional plastic-fiber reference. The final mixture selection should be made according to whether balanced MS–ITS performance, MS-dominant performance, or ITS-dominant performance is desired and should be validated through subsequent experiments.

## 4. Graphical User Interface Platform

**Figure 12:**
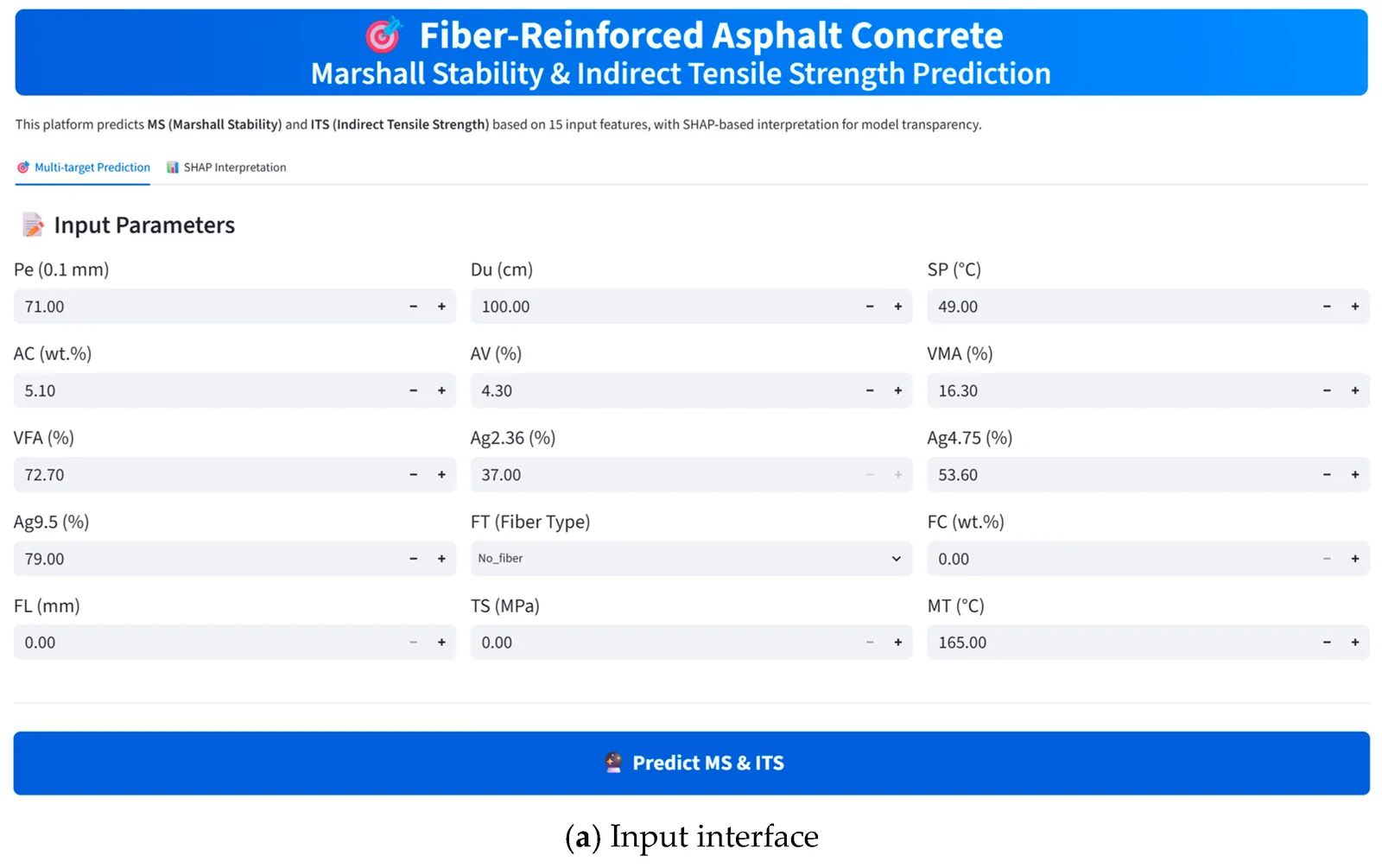

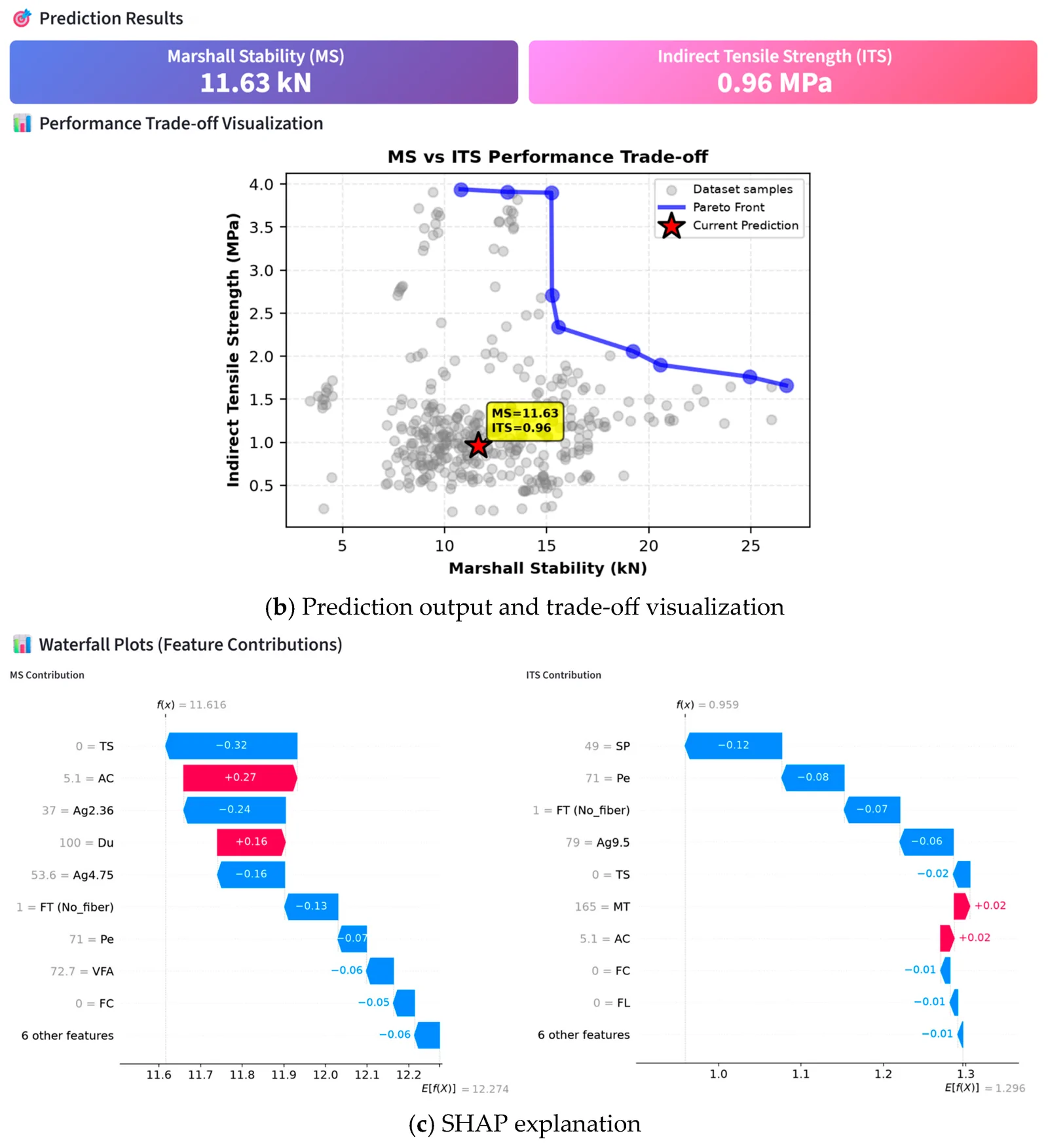
Streamlit-based GUI for MS and ITS prediction and interpretation.

For practical application of the proposed framework, a web-based GUI was developed using Streamlit, as shown in Figure 12, and is available at https://acmsitsgui-35achzf3lbbswu8cjarmmj.streamlit.app/ (accessed on 3 August 2026). After users enter mixture composition, aggregate gradation, volumetric parameters, and fiber-related variables, the platform provides real-time predictions of Marshall Stability (MS) and Indirect Tensile Strength (ITS). The predicted mixture is also displayed in the MS-ITS performance trade-off space relative to the experimental samples and Pareto front, thereby providing an intuitive reference for preliminary asphalt-mixture design [81].

Considering that the GUI is deployed in a CPU-resource-constrained Streamlit Community Cloud environment, Random Forest (RF) was selected as the underlying prediction model for both MS and ITS to balance predictive accuracy, online inference efficiency, and local interpretability. Although TabPFN and TabICLv2 achieved superior overall predictive performance, RF retained good predictive capability with lower CPU inference overhead and enabled efficient local explanations using TreeSHAP. Therefore, RF was considered more suitable for deployment in a real-time interactive GUI; this deployment choice does not alter the performance comparison or ranking of the four models reported above.

## 5. Discussion

### 5.1. Overall Effectiveness

As shown in Table 9, previous machine learning studies have demonstrated the feasibility of predicting asphalt-mixture properties, although direct numerical comparisons remain limited by differences in datasets, input variables, and validation strategies. The main contribution of present study is therefore not only the simultaneous prediction of MS and ITS, but also the integration of prediction with explicit trade-off analysis and decision-oriented mixture screening. The results suggest that tabular models are well suited to small-sample and heterogeneous material datasets, whereas RF and XGBoost remain useful benchmark and deployment models because of their computational efficiency and interpretability. In addition, the combination of Pareto filtering and TOPSIS preserves experimentally observed mixtures with different MS–ITS trade-offs and provides transparent design guidance within the current database, rather than claiming generally optimal mixture proportions.

### 5.2. Challenges and Limitations

Although the machine learning framework constructed in this study shows good effectiveness in the dual-index prediction of MS and ITS and mixture selection, some challenges and limitations still remain. (1) The current database contains 389 experimental samples, and the data scale is relatively limited, with a certain degree of heterogeneity in sample sources. Material sources, test conditions, and preparation processes may differ among different studies. Meanwhile, some important material parameters and test information have not yet been included as model inputs, such as fiber surface treatment methods, aggregate morphology, asphalt modification type, and forming method. This may limit the model’s ability to fully characterize complex material behavior. (2) For non-fiber samples, this study assigned fiber-related variables such as fiber content, fiber length, and tensile strength to 0 to distinguish non-fiber samples from fiber-reinforced samples. Although this treatment helps unify the data format and meet the input requirements of the models, it may also introduce certain encoding correlations among fiber-related variables, thereby affecting the model’s independent identification of fiber effects. (3) The Pareto filtering and TOPSIS methods adopted in this study mainly conduct performance trade-off analysis and candidate mixture selection within the range of existing experimental samples. Therefore, the results should be understood as balanced selections within current database rather than generally optimal mixture proportions. In addition, this study only used Marshall stability and indirect tensile strength as optimization objectives, and has not further considered factors such as moisture stability, fatigue performance, material cost, and environmental impact. Therefore, the current selection results are more suitable used as references for preliminary mix proportion analysis and subsequent experimental validation, rather than directly replacing a complete engineering design and performance evaluation process.

### 5.3. Future Research Perspectives

In response to the above limitations, future research can further improve the current framework from three aspects: data expansion, variable encoding optimization, and multi-objective design extension. First, the scale of the asphalt concrete database should be continuously expanded, and the completeness and consistency of data fields should be improved. In addition to the existing input features, future studies can further include information such as fiber surface treatment methods, aggregate morphology, asphalt modification type, forming method, test temperature, and loading conditions. This would allow the effects of different material compositions and test conditions on MS and ITS to be more comprehensively characterized, and improve the generalization performance of the model on external data and practical engineering scenarios. Second, regarding the differences between non-fiber samples and fiber-reinforced samples, future studies can explore more reasonable feature representation methods, such as introducing an independent fiber-presence variable, adopting group-based modeling strategies, or designing encoding methods that more suitable for mixed-type material data. These methods may reduce the encoding correlations caused by zero assignment of fiber-related variables and enhance the model’s ability to independently identify fiber effects. Finally, in terms of mixture selection and optimization, future studies can further combine machine learning prediction models, engineering constraints, and multi-objective optimization algorithms based on existing observed-data Pareto filtering and TOPSIS analysis, to generate and screen new candidate mix proportions. Meanwhile, the optimization objectives should also be extended from MS and ITS to multiple objectives, including moisture stability, fatigue performance, material cost, and environmental impact. The reliability of the model-recommended results should also be verified through experiments, thereby promoting the development of the current framework from preliminary performance analysis toward a more complete intelligent design method for asphalt mixtures.

## 6. Conclusions

This study constructed a machine learning framework for the dual-parameter prediction of Marshall stability and indirect tensile strength and data-driven mixture optimization for the preliminary design of asphalt concrete. Based on the current research results, the main conclusions are as follows:

- This study established an asphalt concrete database containing 389 experimental samples and selected 15 input variables to characterize factors, such as asphalt properties, aggregate gradation, volumetric parameters, and fiber characteristics. Different from prediction tasks that focus only on a single mechanical property, this study used both MS and ITS as output indicators, providing a data basis for the dual-index performance prediction and subsequent mix proportion selection of asphalt mixtures.
- In terms of model prediction, TabPFN and TabICLv2 demonstrated strong comprehensive predictive performance for small-sample, multivariable tabular data, outperforming the Optuna-optimized RF and XGBoost models overall. For MS prediction, TabICLv2 performed slightly better, achieving an R^2^ of 0.85 ± 0.04 and an RMSE of 1.49 ± 0.22 kN, compared with 0.84 ± 0.04 and 1.53 ± 0.21 kN for TabPFN. For ITS prediction, TabPFN showed a slight advantage, with an R^2^ of 0.91 ± 0.06 and an RMSE of 0.23 ± 0.08 MPa. RF and XGBoost also maintained acceptable predictive accuracy and can serve as reliable benchmark models. Based on the normalized integration of multiple evaluation metrics, TabICLv2 achieved the highest composite score of 0.876, followed closely by TabPFN with 0.859, confirming their comparable and superior overall performance.
- Nine Pareto samples were identified from the 389 valid samples, indicating that only a limited number of observed mixtures simultaneously achieved relatively high MS and ITS. A clear trade-off was observed between the two properties, suggesting that maximizing either indicator alone does not necessarily produce the most balanced mixture. Among the Pareto samples, E122 provided the best compromise under the equal-weight TOPSIS scenario, with an MS of 15.23 kN and an ITS of 3.90 MPa. Based on the characteristics of the Pareto samples, several design-oriented implications were obtained. For balanced MS–ITS performance, low-dosage mineral-fiber systems may be prioritized, with fiber contents of 0.20–0.30%, a fiber length of 6 mm, and asphalt contents of 5.27–5.80%. For MS-oriented design, carbon-fiber systems showed greater potential, corresponding to fiber contents of 0.40–0.80% and asphalt contents of 5.90–6.70%. For ITS-oriented design, the plastic-fiber sample with a fiber content of 0.23% and an asphalt content of 4.00% achieved the highest ITS. In addition, one non-fiber sample remained on the Pareto front, indicating that fiber reinforcement is not the only pathway to achieving a favorable MS–ITS combination.
- To improve the practical usability of the model, this study further developed a graphical user interface platform based on Streamlit. The platform integrates real-time MS and ITS prediction, MS–ITS performance trade-off visualization, and local explanation functions, helping users intuitively evaluate the relative position of the input mix proportion among the experimental samples and the Pareto front and assisting in the understanding of the model prediction results.

## Figures and Tables

**Table 1:** Overview of fiber categories and their amounts.

Fiber Categories	Sample Size
Plastic fiber	110
Mineral fiber	41
Bio-fiber	36
Carbon fiber	19
Glass-fiber	16
Steel-fiber	15
No-fiber	152
Total	389

**Table 2:** Descriptive statistics of input and output variables.

Variable	Unit	Min	Q1	Q2	Q3	Max	Mean	STD
Pe	0.1 mm	37	47	65	71.20	91.80	64.24	14.82
Du	cm	85	100	100	150	168	119.17	24.94
SP	°C	42	48.20	50	51.60	81	51.75	7.09
AC	wt.%	3.16	4.70	5.00	5.50	10.39	5.20	0.82
AV	%	1.60	3.90	4.30	4.99	7.50	4.40	0.98
VMA	%	12.10	15.50	16.34	17.26	32.33	16.61	2.14
VFA	%	54.17	68.19	72.68	75.17	92.50	72.54	5.86
Ag2.36	%	19.45	30.95	37.00	42.84	85.00	36.85	8.34
Ag4.75	%	24.00	46.96	54.00	59.18	95.00	51.66	11.49
Ag9.5	%	42.50	71.21	79.10	83.00	100.00	75.89	11.59
FT	/	/	/	/	/	/	/	/
FC	wt.%	0	0	0.20	0.40	2.25	0.27	0.35
FL	mm	0	0	6.00	12.00	125.00	9.49	20.77
TS	MPa	0	0	0.084	2320	4900	918.97	1329.91
MT	°C	0	0	0	220	1650	125.91	234.66
MS	kN	3.40	9.50	11.70	14.78	26.72	12.26	3.87
ITS	MPa	0.20	0.82	1.10	1.44	3.94	1.29	0.79

**Table 3:** Overview of machine learning models.

No.	Model	Category	Notes
1	TabICLv2	Foundation model	Pretrained transformer for tabular prediction
2	TabPFN	Foundation model	In-context learning for tabular regression
3	RF	Ensemble—Bagging	Bagging of decision trees
4	XGBoost	Ensemble—Boosting	Boosting model with regularization

**Table 4:** Model configurations and optimized hyperparameter settings.

Models	Hyperparameters
TabICLv2	n_estimators = 8; feat_shuffle = Latin hypercube; outlier_threshold = 4.0; batch_size = 8; device = CUDA
TabPFN	n_estimators = 8; auto_scale = True; softmax_temperature = 0.9; inference_precision = auto; device = CUDA
RF	n_estimators = 175; max_depth = 19; min_samples_split = 2; min_samples_leaf = 1; max_features = sqrt; bootstrap = True; n_jobs = −1
XGBoost	n_estimators = 718; max_depth = 9; learning_rate = 0.025; reg_alpha = 0.24; reg_lambda = 1.11; min_child_weight = 9.68; objective = reg:squarederror; tree_method = hist; n_jobs = −1

**Table 5:** Model performance summary over 50 Monte Carlo splits.

Model	RMSE	MAE	MAPE (%)	MAD	R^2^	Time (s/Split)
TabICLv2	MS: 1.49 ± 0.22	MS: 1.03 ± 0.11	MS: 8.68 ± 1.18	MS: 0.72 ± 0.11	MS: 0.85 ± 0.04	1.45 ± 0.05
	ITS: 0.24 ± 0.08	ITS: 0.13 ± 0.03	ITS: 11.75 ± 3.23	ITS: 0.07 ± 0.01	ITS: 0.90 ± 0.07	
TabPFN	MS: 1.53 ± 0.21	MS: 1.06 ± 0.12	MS: 8.84 ± 1.24	MS: 0.72 ± 0.11	MS: 0.84 ± 0.04	3.39 ± 0.23
	ITS: 0.23 ± 0.08	ITS: 0.13 ± 0.03	ITS: 10.92 ± 2.76	ITS: 0.07 ± 0.01	ITS: 0.91 ± 0.06	
RF	MS: 1.61 ± 0.24	MS: 1.15 ± 0.14	MS: 10.02 ± 1.61	MS: 0.82 ± 0.12	MS: 0.82 ± 0.05	0.27 ± 0.02
	ITS: 0.27 ± 0.05	ITS: 0.17 ± 0.03	ITS: 18.40 ± 5.31	ITS: 0.10 ± 0.02	ITS: 0.88 ± 0.05	
XGBoost	MS: 1.63 ± 0.20	MS: 1.16 ± 0.12	MS: 9.73 ± 1.24	MS: 0.80 ± 0.10	MS: 0.81 ± 0.04	1.26 ± 0.13
	ITS: 0.27 ± 0.06	ITS: 0.16 ± 0.02	ITS: 16.08 ± 5.42	ITS: 0.09 ± 0.01	ITS: 0.88 ± 0.06	

Note: RF and XGBoost were evaluated on an Intel Core i7-12800HX CPU; TabPFN and TabICLv2 were evaluated on an NVIDIA GeForce RTX 4070 Laptop GPU (8 GB).

**Table 6:** Sensitivity analysis of TabPFN performance under pooled and separated fiber-status modeling strategies.

Target	Test Subset	Pooled Encoding R^2^	Separated Models R^2^	Pooled Encoding RMSE	Separated Models RMSE	Wilcoxon *p*-Value
MS	All samples	0.837 ± 0.043	0.795 ± 0.062	1.529 ± 0.209 kN	1.710 ± 0.268 kN	<0.001
	Fiber-only	0.870 ± 0.047	0.851 ± 0.075	1.336 ± 0.236 kN	1.411 ± 0.321 kN	0.004
	Non-fiber-only	0.770 ± 0.085	0.690 ± 0.116	1.767 ± 0.304 kN	2.056 ± 0.404 kN	<0.001
ITS	All samples	0.903 ± 0.069	0.877 ± 0.083	0.231 ± 0.078 MPa	0.261 ± 0.080 MPa	<0.001
	Fiber-only	0.914 ± 0.081	0.911 ± 0.087	0.240 ± 0.104 MPa	0.243 ± 0.101 MPa	0.126
	Non-fiber-only	0.817 ± 0.111	0.646 ± 0.195	0.195 ± 0.072 MPa	0.273 ± 0.085 MPa	<0.001

Note: Values are reported as mean ± standard deviation across 50 matched Monte Carlo train–test splits. The *p*-values were obtained from two-sided paired Wilcoxon signed-rank tests based on split-wise RMSE values. RMSE is expressed in kN for MS and MPa for ITS.

**Table 7:** Information and TOPSIS ranking of the observed-data Pareto solutions.

Feature	E122	E112	E263	E265	E239	E266	E267	E069	E126
Rank	1	2	3	4	5	6	7	8	9
FT	Mineral fiber	Mineral fiber	Carbon fiber	Carbon fiber	Plastic fiber	Carbon fiber	Carbon fiber	No-fiber	Plastic fiber
Pe	91.6	91.8	60	60	85.9	60	60	47	57
Du	150.00	150.00	100.00	100.00	100.00	100.00	100.00	100.00	NA
SP	46.90	49.60	42.00	42.00	46.50	42.00	42.00	61.00	51.60
AC	5.27	5.80	6.20	6.50	4.00	5.90	6.70	4.30	4.65
AV	3.10	3.36	4.89	5.29	3.81	7.01	6.08	3.90	4.41
VMA	14.20	16.75	14.84	15.19	NA	17.69	15.90	12.10	NA
VFA	78.60	79.94	67.03	65.20	NA	60.35	61.78	67.77	NA
Ag2.36	33.90	37.00	37.70	37.70	23.00	37.70	37.70	26.60	39.06
Ag4.75	54.80	53.00	54.00	54.00	33.50	54.00	54.00	37.90	48.63
Ag9.5	80.90	76.50	79.10	79.10	55.50	79.10	79.10	58.30	76.27
FC	0.20	0.30	0.40	0.60	0.23	0.80	0.80	0.00	0.075
FL	6	6	6	6	6	6	6	0	4
TS	2320	2320	3500	3500	591	3500	3500	0	780
MT	NA	NA	NA	NA	259	NA	NA	0	NA
MS	15.23	13.06	26.72	24.95	10.78	20.55	19.22	15.26	15.56
ITS	3.90	3.91	1.66	1.76	3.94	1.90	2.06	2.71	2.34
TOPSIS score	0.572	0.522	0.516	0.496	0.484	0.402	0.380	0.370	0.299

**Table 8:** Design-oriented summary of observed-data Pareto solutions.

Design Pathway	Representative Pareto Solutions	Fiber System	FC (%)	FL (mm)	AC (%)	MS (kN)	ITS (MPa)	Design Implication
Balanced MS–ITS	E122, E112	Mineral fiber (basalt)	0.20–0.30	6.00	5.27–5.80	13.06–15.23	3.90–3.91	Candidate window for balanced MS–ITS performance
MS-dominant	E263, E265, E266, E267	Carbon fiber (carbon)	0.40–0.80	6.00	5.90–6.70	19.22–26.72	1.66–2.06	High MS can be achieved, but with a lower ITS level
ITS-dominant	E239	Plastic fiber (polyester)	0.23	6.00	4.00	10.78	3.94	Highest ITS, although MS compensation may be required
No-fiber reference	E069	No fiber	0.00	0.00	4.30	15.26	2.71	Non-fiber Pareto baseline
Plastic-fiber reference	E126	Plastic fiber (polyacrylonitrile)	0.075	4.00	4.65	15.56	2.34	Lower-ranked plastic-fiber Pareto option

**Table 9:** Comparison of related ML prediction studies and the present study.

Study	Dataset Scale	Prediction Target	Best Model	Best Reported Predictive Performance
[19]	ABC: *n* = 253; AWC: *n* = 343; 8 inputs	Marshall stability and Marshall flow value	MEP	MS: R^2^ = 0.81–0.94, RMSE = 43.32–46.71 kg; Marshall flow: R^2^ = 0.92–0.94, RMSE = 0.41–0.90.
[20]	17,920 raw records; final *n* = 2013 for HWT and *n* = 1866 for ITS	Hamburg wheel tracking rut depth and Indirect tensile strength	Extra Trees	HWT rut depth: R^2^ = 0.92; ITS: R^2^ = 0.90.
[26]	*n* = 2490; 15 inputs; 6 fiber categories	Marshall stability, Marshall flow value, and Marshall quotient	Voting model	MS: R^2^ = 0.89, RMSE = 1.51; flow: R^2^ = 0.85, RMSE = 1.16; Marshall quotient: R^2^ = 0.88, RMSE = 0.60.
[27]	*n* = 296; 14 inputs	Splitting strength	TabPFN	R^2^ = 0.85 ± 0.08; RMSE = 0.34 ± 0.10 MPa.
[29]	*n* = 732; 8 inputs	Marshall stability and Marshall flow value	RF	MS: R^2^ = 0.93, RMSE = 1.70; Marshall flow: R^2^ = 0.81, RMSE = 0.36.
Present study	*n* = 389; 15 inputs	Marshall stability and Indirect tensile strength	TabICLv2 for MS; TabPFN for ITS	MS: R^2^ = 0.85 ± 0.04, RMSE = 1.49 ± 0.22 kN; ITS: R^2^ = 0.91 ± 0.06, RMSE = 0.23 ± 0.08 MPa.

Note: *n* denotes the dataset size.

**Figure A1:**
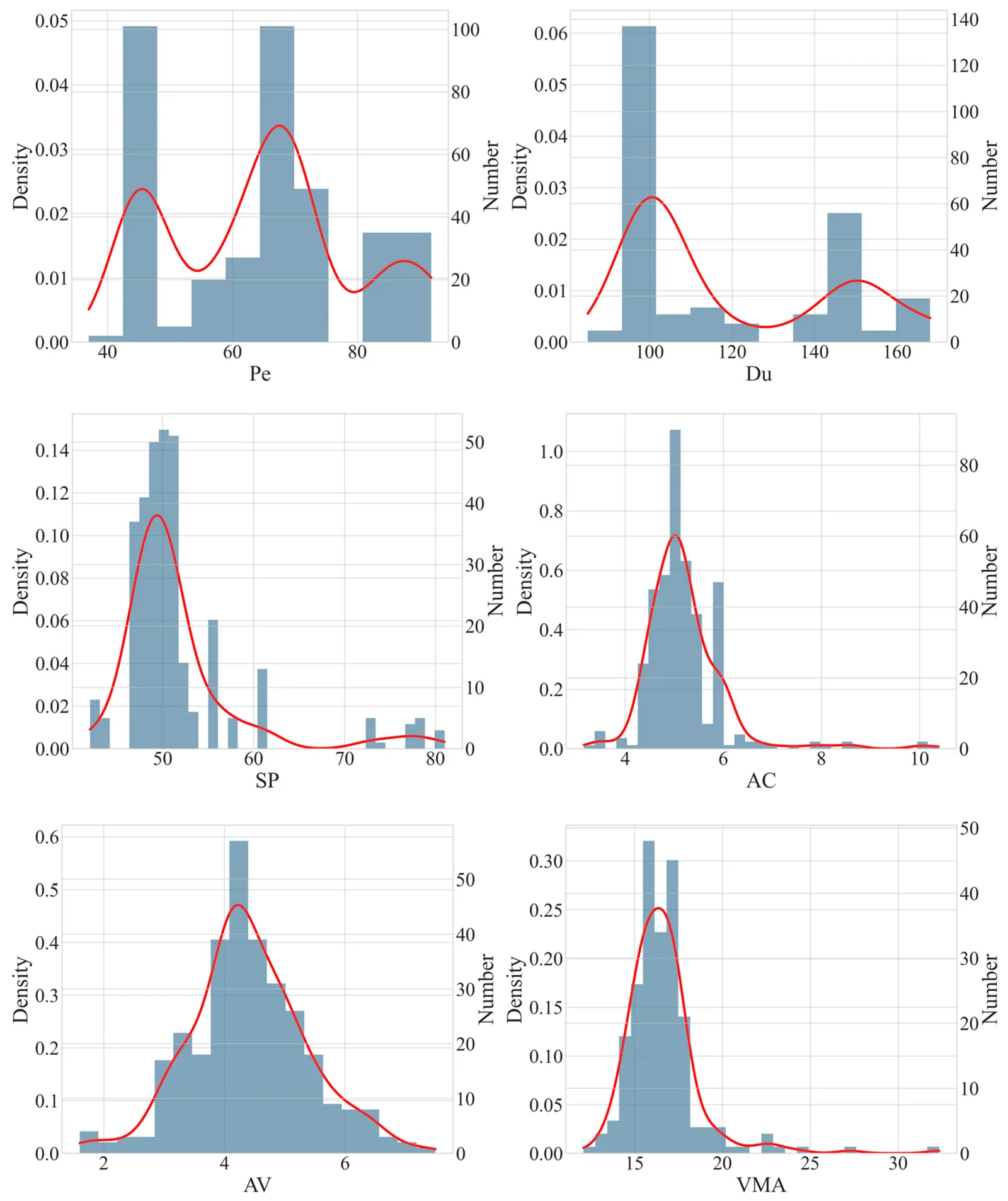

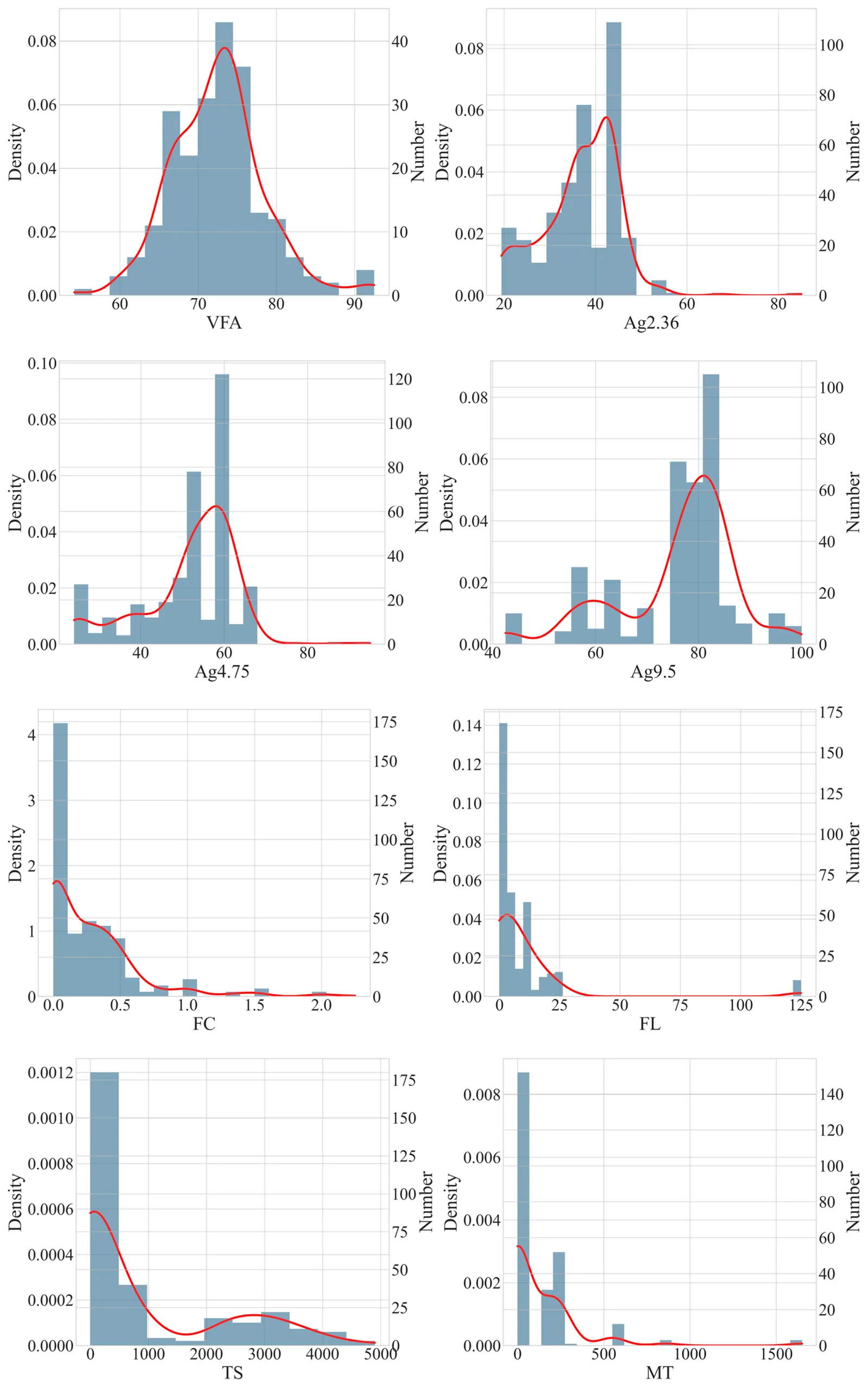

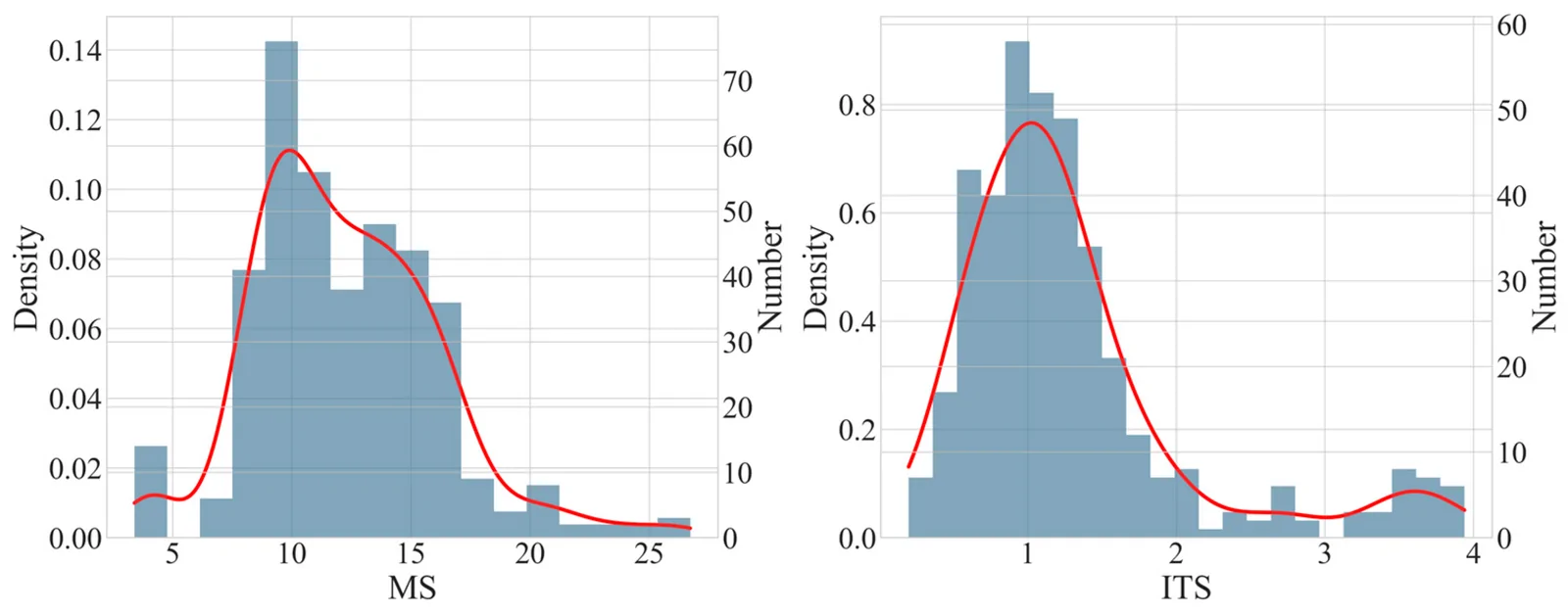
Distribution characteristics of dataset features.

## Data Availability

The original contributions presented in this study are included in the article. Further inquiries can be directed to the corresponding authors.

## Abbreviations

Abbreviation	Full name
GUI	Graphical User Interface
ITS	Indirect Tensile Strength
MAD	Median Absolute Deviation
MAE	Mean Absolute Error
MAPE	Mean Absolute Percentage Error
MS	Marshall Stability
R^2^	Coefficient of Determination
RF	Random Forest
RMSE	Root Mean Square Error
SHAP	SHapley Additive exPlanations
TabICLv2	Tabular In-Context Learning version 2
TabPFN	Tabular Prior-data Fitted Network
TOPSIS	Technique for Order Preference by Similarity to Ideal Solution
XGBoost	Extreme Gradient Boosting

## Appendix A. Software Environment and Dataset Characteristics

**Table A1:** Computational setup and key Python packages.

Category	Software/Library	Version
Programming environment	Python	3.13.12
Numerical computing	NumPy	2.4.3
Data processing	pandas	3.0.1
Machine learning	scikit-learn	1.6.1
Machine learning	XGBoost	3.2.0
Foundation model	TabPFN	8.0.8
Foundation model	TabICLv2/tabicI	2.1.1
Deep learning backend	PyTorch	2.6.0+cu124
Explainable AI	SHAP	0.52.0
Visualization	matplotlib	3.10.9
Model persistence	joblib	1.5.3
GUI development	Streamlit	1.58.0

## Appendix B. Model Settings and Prediction Performance

**Table A2:** Optuna Search Space.

Model	Parameter	Distribution	Search Range	Selected Value
RF	n_estimators	Int (uniform)	100–700	175
	max_depth	Int (uniform)	3–30	19
	min_samples_split	Int (uniform)	2–12	2
	min_samples_leaf	Int (uniform)	1–6	1
	max_features	Categorical	{sqrt, log_2_, 1.0}	sqrt
XGBoost	n_estimators	Int (uniform–)	100–800	718
	max_depth	Int (uniform)	2–10	9
	learning_rate	Float (log-uniform)	10^−3^–0.1	0.025
	reg_alpha	Float (log-uniform)	10^−4^–10	0.24
	reg_lambda	Float (log-uniform)	10^−4^–10	1.11
	min_child_weight	Float (uniform)	1–10	9.68

**Table A3:** Formal paired comparison of model performance across 50 Monte Carlo splits.

Target	Metric	Omnibus Friedman *p*	TabPFN vs. RF	TabPFN vs. XGBoost	TabICLv2 vs. RF	TabICLv2 vs. XGBoost
MS	RMSE ↓	2.31 × 10^−10^	8.48 × 10^−5^ * (TabPFN)	1.52 × 10^−5^ * (TabPFN)	1.80 × 10^−10^ * (TabICLv2)	1.17 × 10^−8^ * (TabICLv2)
	MAPE (%) ↓	5.59 × 10^−21^	6.22 × 10^−13^ * (TabPFN)	9.40 × 10^−10^ * (TabPFN)	1.07 × 10^−14^ * (TabICLv2)	2.18 × 10^−12^ * (TabICLv2)
	MAE ↓	1.57 × 10^−19^	2.94 × 10^−9^ * (TabPFN)	1.55 × 10^−9^ * (TabPFN)	1.07 × 10^−14^ * (TabICLv2)	1.78 × 10^−14^ * (TabICLv2)
	R^2^ ↑	2.31 × 10^−10^	1.46 × 10^−4^ * (TabPFN)	3.18 × 10^−5^ * (TabPFN)	2.98 × 10^−10^ * (TabICLv2)	1.85 × 10^−8^ * (TabICLv2)
	MAD ↓	1.89 × 10^−14^	5.79 × 10^−8^ * (TabPFN)	8.50 × 10^−6^ * (TabPFN)	1.34 × 10^−11^ * (TabICLv2)	7.44 × 10^−6^ * (TabICLv2)
ITS	RMSE ↓	2.93 × 10^−7^	5.43 × 10^−7^ * (TabPFN)	1.32 × 10^−4^ * (TabPFN)	4.99 × 10^−6^ * (TabICLv2)	2.71 × 10^−4^ * (TabICLv2)
	MAPE (%) ↓	5.83 × 10^−27^	1.07 × 10^−14^ * (TabPFN)	1.78 × 10^−14^ * (TabPFN)	1.78 × 10^−14^ * (TabICLv2)	1.01 × 10^−13^ * (TabICLv2)
	MAE ↓	9.33 × 10^−24^	1.07 × 10^−14^ * (TabPFN)	3.06 × 10^−13^ * (TabPFN)	4.44 × 10^−14^ * (TabICLv2)	7.30 × 10^−13^ * (TabICLv2)
	R^2^ ↑	2.93 × 10^−7^	1.05 × 10^−5^ * (TabPFN)	5.21 × 10^−4^ * (TabPFN)	4.44 × 10^−5^ * (TabICLv2)	1.65 × 10^−3^ * (TabICLv2)
	MAD ↓	2.27 × 10^−20^	1.07 × 10^−14^ * (TabPFN)	8.09 × 10^−11^ * (TabPFN)	1.24 × 10^−13^ * (TabICLv2)	1.31 × 10^−10^ * (TabICLv2)

Note: Omnibus *p*-values were obtained using the Friedman test across the four models. Pairwise *p*-values were obtained using two-sided Wilcoxon signed-rank tests with Holm correction. The model shown in parentheses indicates the better-performing model. * Adjusted *p* < 0.05. Arrows indicate the preferred direction of each metric.
